# Gain of Function Mutations in *CgPDR1* of *Candida glabrata* Not Only Mediate Antifungal Resistance but Also Enhance Virulence

**DOI:** 10.1371/journal.ppat.1000268

**Published:** 2009-01-16

**Authors:** Sélène Ferrari, Françoise Ischer, David Calabrese, Brunella Posteraro, Maurizio Sanguinetti, Giovanni Fadda, Bettina Rohde, Christopher Bauser, Oliver Bader, Dominique Sanglard

**Affiliations:** 1 Institute of Microbiology, University of Lausanne and University Hospital Center, Lausanne, Switzerland; 2 Institute of Microbiology, Università Cattolica del Sacro Cuore, Rome, Italy; 3 GATC Biotech AG, Konstanz, Germany; 4 Institut für Medizinische Mikrobiologie, Universitätskliniken Göttingen, Göttingen, Germany; David Geffen School of Medicine at University of California Los Angeles, United States of America

## Abstract

CgPdr1p is a *Candida glabrata* Zn(2)-Cys(6) transcription factor involved in the regulation of the ABC-transporter genes *CgCDR1*, *CgCDR2*, and *CgSNQ2*, which are mediators of azole resistance. Single-point mutations in *CgPDR1* are known to increase the expression of at least *CgCDR1* and *CgCDR2* and thus to contribute to azole resistance of clinical isolates. In this study, we investigated the incidence of *CgPDR1* mutations in a large collection of clinical isolates and tested their relevance, not only to azole resistance *in vitro* and *in vivo*, but also to virulence. The comparison of *CgPDR1* alleles from azole-susceptible and azole-resistant matched isolates enabled the identification of 57 amino acid substitutions, each positioned in distinct *CgPDR1* alleles. These substitutions, which could be grouped into three different “hot spots,” were gain of function (GOF) mutations since they conferred hyperactivity to CgPdr1p revealed by constitutive high expression of ABC-transporter genes. Interestingly, the major transporters involved in azole resistance (*CgCDR1*, *CgCDR2*, and *CgSNQ2*) were not always coordinately expressed in presence of specific *CgPDR1* GOF mutations, thus suggesting that these are rather *trans*-acting elements (GOF in *CgPDR1*) than *cis*-acting elements (promoters) that lead to azole resistance by upregulating specific combinations of ABC-transporter genes. Moreover, *C. glabrata* isolates complemented with *CgPDR1* hyperactive alleles were not only more virulent in mice than those with wild type alleles, but they also gained fitness in the same animal model. The presence of *CgPDR1* hyperactive alleles also contributed to fluconazole treatment failure in the mouse model. In conclusion, this study shows for the first time that *CgPDR1* mutations are not only responsible for *in vitro*/*in vivo* azole resistance but that they can also confer a selective advantage under host conditions.

## Author Summary

*Candida glabrata* is a yeast causing several diseases in humans and especially in immuno-compromised people. *C. glabrata* infections are treated with antifungal agents, however the use of some agents, for example azoles, is associated with the development of resistance. In this yeast species, azole resistance is mediated almost exclusively by ATP Binding Cassette (ABC) multidrug transporters. Their overexpression results in enhanced efflux of azoles and thus generates resistance. Regulation of ABC transporters is therefore of pivotal importance to understanding azole resistance. In *C. glabrata*, the expression of ABC transporters is mediated by a zinc finger transcription factor called *CgPDR1*. Gain of function (GOF) mutations in *CgPDR1* result in high ABC transporter expression. In this study, we investigated the occurrence of GOF mutations in a large collection of azole-resistant isolates and found a high variety of mutations localized in three distinct domains of *CgPDR1*. We found that these mutations are not only associated with resistance but also enhanced virulence and fitness of *C. glabrata* in animal models. Our study provides for the first time evidence that mutations causing antifungal resistance can also provide a selective advantage under host conditions and thus highlights the need of carefully monitoring resistance in this pathogen.

## Introduction

*Candida glabrata* has recently emerged as the second most common cause of invasive candidiasis, and there are increasing numbers of reports showing its important role in mucosal or bloodstream infections [1],[2]. Systemic infections due to *C. glabrata* are characterized by a high mortality rate, and they are difficult to treat due to the intrinsically low susceptibility of this species to azole drugs, especially to fluconazole [3]. In addition, *C. glabrata* easily develops fluconazole resistance in response to drug exposure during patient treatment [4]–[6].

Azole antifungals target the cytochrome P-450 lanosterol 14-α demethylase, encoded by *ERG11*. Resistance of yeast clinical isolates to azole antifungal agents can result from either overexpression or mutations in *ERG11*. Alternatively, the cells can fail to accumulate azole antifungal agents due to enhanced drug efflux, a consequence of transcriptional activation of drug efflux pumps (for review, see [7]). At least two families of multidrug transporters, the ABC (ATP-binding cassette) transporter family and the major facilitator superfamily (MFS), are involved in azole resistance. In *C. glabrata*, the constitutive upregulated expression of ABC-transporter genes *CgCDR1* and, to a lesser extent, *CgCDR2* (also known as *PDH1*) plays a dominant role in azole resistance [4], [8]–[11]. Each of these genes can be upregulated in *C. glabrata* clinical isolates and disruption of *CgCDR1* or *CgCDR1/CgCDR2* leads to hypersusceptibility to fluconazole, cycloheximide, and chloramphenicol [4],[9],[10],[12],[13].

The expression of *CgCDR* genes is regulated by a single Zn(2)-Cys(6) transcription factor, CgPdr1p, an homologue of *S. cerevisiae* Pdr1p/Pdr3p [11]. *CgPDR1* deletion leads to a loss of *CgCDR1* and *CgCDR2* regulation and to a sharp increase in azole susceptibility [14]. Due to the presence of PDRE (pleiotropic drug response element) sequences in the *CgCDR1* and *CgCDR2* promoters, CgPdr1p acts probably by binding to these regulatory elements as Pdr1p and Pdr3p in *S. cerevisiae*. *CgPDR1* contains a PDRE in its promoter suggesting an auto-regulation of its transcription. Consistent with this observation, upregulation of *CgCDR1* and *CgCDR2* in azole-resistant strains is correlated with an increase of *CgPDR1* expression [14],[15]. *CgPDR1* is also essential in azole resistance caused by mitochondrial dysfunction in *C. glabrata* petite mutants. Since enhanced *CgPDR1* expression is observed in some petite mutants, it has been proposed that *CgPDR1* regulates its own expression in response to mitochondrial dysfunction [15]. CgPdr1p acts as nuclear receptor by directly binding to diverse drugs and xenobiotics, such as azoles, to activate expression of efflux pumps genes resulting in multidrug resistance [16]. The activation domain of CgPdr1p binds directly to the Mediator co-activator subunit CgGal11p in a xenobiotic-dependent manner in order to activate transcription of target genes [16].

Two studies have identified three separate amino acid substitutions (W297S, F575L, P927L) in CgPdr1p of azole-resistant strains that are responsible for constitutive high expression of *CgCDR1*, *CgCDR2* and *CgPDR1* itself [14],[15]. Recently, another Pdr1p-regulated ABC-transporter gene, *CgSNQ2*, was shown to participate to azole resistance of *C. glabrata* clinical isolates [17]. In this study, a fourth CgPdr1p amino substitution, P822L, was identified. Interestingly, the P822L substitution is responsible for the constitutive overexpression of *CgSNQ2*, but has no effect on the expression of *CgCDR1* and *CgCDR2* [17].

In the present study, we investigated the incidence of *CgPDR1* mutations in a large collection of clinical isolates. Because no study has yet addressed whether the presence of *CgPDR1* mutations is correlated with fitness costs in *C. glabrata*, we engineered isogenic strains with individual *CgPDR1* mutations and tested their virulence in two different animal models. The strains that acquired *in vitro* azole resistance were used to test the *in vivo* response to fluconazole. We observed a high diversity among *CgPDR1* alleles and identified 57 distinct single amino acid substitutions, which may confer hyperactivity to CgPdr1p in order to mediate high expression of ABC transporter genes. Although *CgCDR1*, *CgCDR2* and *CgSNQ2* are all regulated by CgPdr1p, they are not always coordinately expressed in azole-resistant isolates indicating that ABC transporter genes were differentially regulated depending on the mutation present on the *CgPDR1* allele. Finally, the identified amino acid substitutions in CgPdr1p enhanced virulence and led to fluconazole treatment failure in mouse models. Taken together our data demonstrate a high variability in *CgPDR1* mutations, which themselves have differentiated effects on target genes including ABC-transporters and probably on yet unidentified virulence factors.

## Results

### Isolation and Characterization of *CgPDR1* Alleles from *C. glabrata* Clinical Isolates

**Figure 1:**
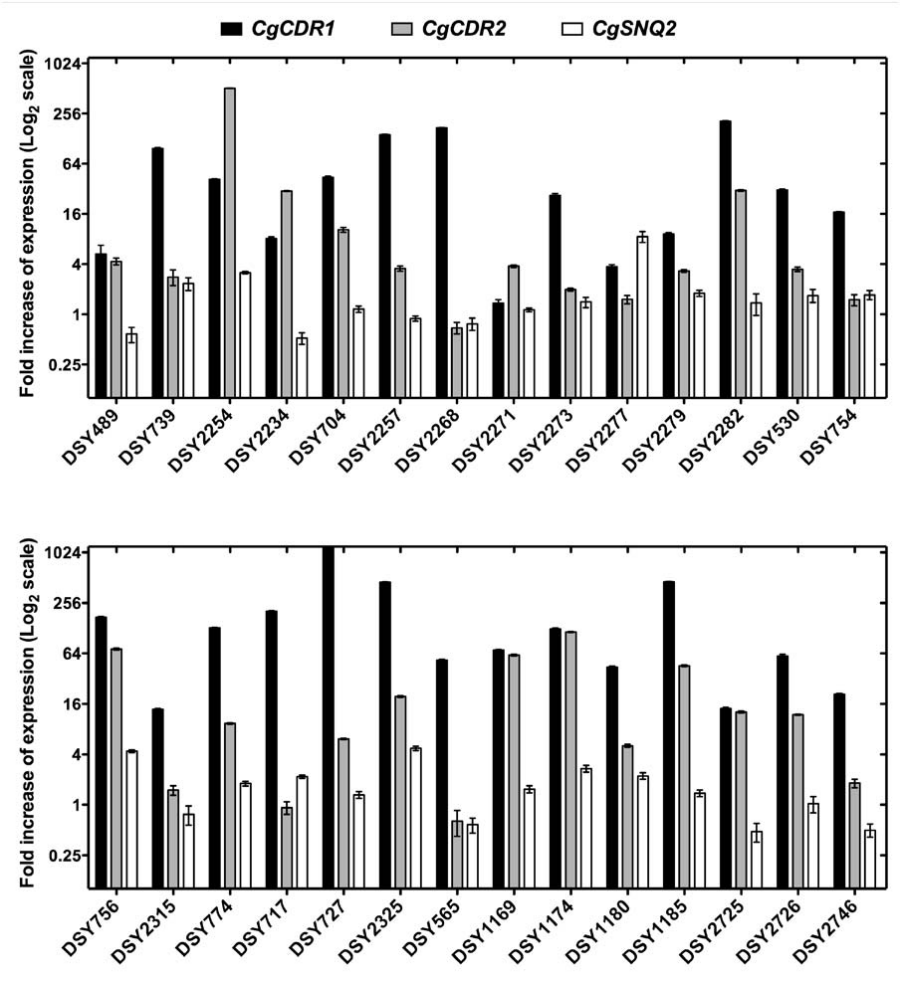
Expression of *CgCDR1*, *CgCDR2*, and *CgSNQ2* in *C. glabrata* azole-resistant isolates from groups of sequential clinical isolates. Quantification was performed by real-time RT-PCR. The values, which are averages of four separated experiments, represent the increase in gene expression relative to the azole-susceptible parental strains (set at 1.00). Error bars show standard deviations.

**Figure 2:**
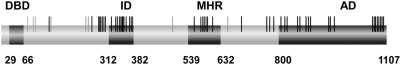
Localisation of 65 non-synonymous substitutions in CgPdr1p. The eight polymorphisms present in *CgPDR1* alleles from both azole-susceptible and azole-resistant isolates are indicated by grey bars. The 57 amino acid polymorphisms (from a total of 58 mutations) specific for *CgPDR1* alleles from azole-resistant isolates are indicated by black bars. Putative DBD (DNA binding domain) and MHR (middle homology region) were located by Pfam analysis. ID (putative inhibitory domain) and AD (putative transcriptional activation domain) were deduced by similarity with Pdr1p and Pdr3p from *S. cerevisiae* [11]. The limits of these domains are indicated by amino acids position in CgPdr1p.

The incidence of *CgPDR1* mutations was investigated in a collection of *C. glabrata* clinical isolates (n = 122) consisting of 30 groups of sequential isolates (n = 66). Each group contained at least one azole-susceptible (MIC fluconazole≤16 µg/ml) and one azole-resistant (MIC fluconazole≥32 µg/ml) isolates, which were shown to be highly related by genotyping methods (Cg6 and Cg12 repetition probes or MLST) (data not shown). There were 36 azole-resistant isolates among the groups of related isolates. The 56 remaining isolates were unrelated (41 azole-resistant and 15 azole-susceptible, Table S1). In this collection, azole-resistant isolates upregulated at least one of the ABC-transporter genes including *CgCDR1*, *CgCDR2* or *CgSNQ2* (see Figure 1 for ABC-transporter genes expression levels measured by real-time RT-PCR in groups of isolates and Figure S1 for CgCdr1p and CgCdr2p levels determined by western blot). *CgPDR1* from each isolate was cloned and sequenced. To determine nucleotide polymorphisms, the 122 sequences were aligned and showed 66 non-synonymous nucleotide substitutions among a total of 70 distinct *CgPDR1* alleles. By comparison of *CgPDR1* alleles from azole-susceptible and azole-resistant isolates, we identified 12 different alleles recovered only from azole-susceptible isolates containing combinations of eight different mutations and 58 different alleles specific for azole-resistant isolates with 58 distinct mutations. These mutations yielded 57 single amino acid substitutions (Table S2) located at 50 locations along the protein and encompassing three distinct protein domains: i) the region similar to the transcriptional inhibitory domain of Pdr1p from *S. cerevisiae*, ii) the middle homology region (MHR) and iii) a putative transcriptional activation domain (Figure 2). Eleven distinct amino acids substitutions were found repeatedly in azole-resistant resistant isolates (six found twice and five found three times) and several substitutions occurred at the same position in six different cases (Table S2). Overall, CgPdr1p unique substitutions were found in 46 distinct azole-resistant isolates. This suggests that saturation of *CgPDR1* mutations in azole-resistant isolates may be still not reached. Indeed, a parallel and independent *CgPDR1* sequence analysis of ten azole-resistant *C. glabrata* isolates still revealed one unknown mutation and two additional distinct mutations on one of the 51 nucleotide positions identified in this study (O. Bader, unpublished). We hypothesized that *CgPDR1* mutations may confer enhanced activity (or hyperactivity) to CgPdr1p leading to increased expression of the *CgCDR* and/or *CgSNQ2* genes.

Azole resistance was generally correlated with the occurrence of mutations in *CgPDR1*, except in four azole-resistant isolates (DSY717, DSY2282, DSY2325 and BPY41). Mitochondrial dysfunction is one of the possible mechanism by which azole resistance can occur during azole treatment of patients [18]. Indeed, three isolates (DSY2282, DSY2325 and BPY41) displayed altered mitochondrial respiratory capacity as deduced from their inability to grow on medium containing glycerol as sole carbon source and from their defects in mitochondrial DNA (Figure S2). Although *CgCDR1* was upregulated in the remaining isolate DSY717, no *CgPDR1* mutation could be detected. We are currently investigating azole resistance in this isolate and these data will be reported elsewhere.

### *CgPDR1* Expression Levels Have a Moderate Effect on Azole Resistance

**Figure 3:**
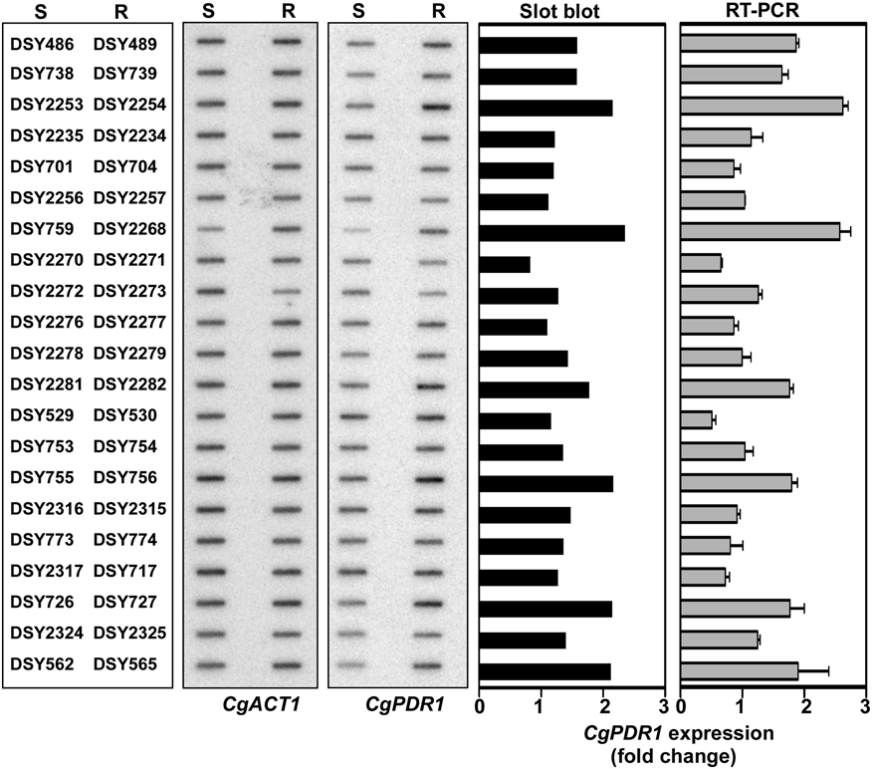
Expression of *CgPDR1* in matched pairs of azole-susceptible (S) and azole-resistant (R) isolates. RNA was isolated from log phase cultures, slot-blotted to membranes and hybridized with the indicated gene probes. *CgACT1* served as internal control. Signals obtained in blotted membranes were quantified by counting radioactivity by phosphor imaging. Signals obtained for *CgPDR1* were normalized with *CgACT1*. Expression values represent the increase of *CgPDR1* expression in azole-resistant isolates relative to their azole-susceptible parental strains. RNA was also used for real-time quantitative RT-PCR as described in Material and Methods. Values given on the right diagram are means (±SEM) of three separate experiments and express *CgPDR1* relative expression in resistant isolates as compared to their matched susceptible parents.

Single point mutations in *CgPDR1* have been shown to increase the expression of both *CgCDR* genes and *CgPDR1*, thus contributing to azole resistance of clinical isolates [14],[15]. To evaluate whether the expression level of *CgPDR1* is important for the upregulation of *CgCDR* genes in our isolates, the *CgPDR1* mRNA levels of 21 matched pairs of azole-susceptible and azole-resistant isolates (listed in Table 1) were quantified by slot blot analysis and real-time RT-PCR (Figure 3). The comparison between *CgPDR1* expression levels from azole-susceptible and azole-resistant matched isolates yielded comparable results as judged by similar relative increase of *CgPDR1* expression obtained with the two methods (Figure 3). We concluded from these results that *CgPDR1* upregulation was not correlating with azole resistance since *CgPDR1* was overexpressed up to two-fold in some resistant isolates (DSY2268, DSY565, DSY756) as compared to their matched azole-susceptible isolate, whereas it was similarly expressed in others (DSY2257, DSY2277, DSY2271) (Figure 3).

**Table 1:** Azole susceptibilities and *CgPDR1* mutations from *C. glabrata* clinical isolates.

Strain	Site of isolation	*CgPDR1* mutation^b^	MIC (µg ml^−1^)^a^		
			FLC^c^	ITC^c^	KTC^c^
DSY486	Oropharynx	-	16	1	1
DSY489	Oropharynx	L328F (G984T)	128	2	2
DSY738	Oropharynx	-	8	0.5	1
DSY739	Oropharynx	R376W (C1126T)	64	2	2
DSY2253	Oropharynx	-	16	1	1
DSY2254	Oropharynx	D1082G (A3245G)	128	2	2
DSY2235	Oropharynx	-	4	0.125	0.125
DSY2234	Oropharynx	T588A (A1762G)	32	1	1
DSY701	Oropharynx		4	0.25	0.25
DSY704	Oropharynx	T607S (C1820G)	32	1	1
DSY529	Oropharynx	-	4	0.5	0.5
DSY530	Oropharynx	E1083Q (G3247C)	64	2	2
DSY753	Oropharynx	-	4	0.125	0.125
DSY754	Oropharynx	Y584C (A1751G)	32	1	1
DSY726	Oropharynx	-	8	0.25	0.25
DSY727	Oropharynx	D876Y (G2626T)	64	2	2
DSY562	Oropharynx	-	8	0.125	0.125
DSY565	Oropharynx	I280F (G840C)	128	2	2
DSY2256	Oropharynx	-	4	2	1
DSY2257	Oropharynx	N691D (A2071G)	32	1	1
DSY759	Oropharynx	-	4	0.5	0.125
DSY2268	Oropharynx	S316I (G947T)	32	1	1
DSY2270	Oropharynx	-	8	1	1
DSY2271	Oropharynx	D261G (A782G)	128	2	2
DSY2272	Oropharynx	-	4	0.5	0.5
DSY2273	Oropharynx	R293I (G878T)	32	2	1
DSY2276	Oropharynx	-	8	0.5	0.5
DSY2277	Oropharynx	R592S (G1776C)	32	2	1
DSY2278	Oropharynx	-	4	0.5	0.5
DSY2279	Oropharynx	G583S (G1747A)	32	0.5	0.5
DSY2281	Oropharynx	-	8	0.25	0.25
DSY2282	Oropharynx	-	64	2	1
DSY755	Oropharynx	-	4	0.25	0.125
DSY756	Oropharynx	S343F (C1028T)	128	2	2
DSY2316	Oropharynx	-	8	1	1
DSY2315	Oropharynx	R376G (C1126G)	32	1	1
DSY773	Oropharynx	-	16	0.5	1
DSY774	Oropharynx	R376G (C1126G)	32	1	1
DSY2317	Oropharynx	-	4	0.25	0.5
DSY717	Oropharynx	-	64	2	2
DSY2324	Oropharynx	-	4	0.25	0.125
DSY2325	Oropharynx	-	128	2	2

a MICs to these antifungal agents were determined by broth microdilution method in accordance with the CLSI M27-A2 document (National Committee for Clinical Laboratory Standards, 2002).

b Numbers correspond to the positions of amino acid changes while numbers in parentheses correspond to the position of changed nucleotides changed in *CgPDR1* relative to the ATG start codon.

c FLC: Fluconazole; ITC: itraconazole; KTC: ketoconazole.

**Figure 4:**
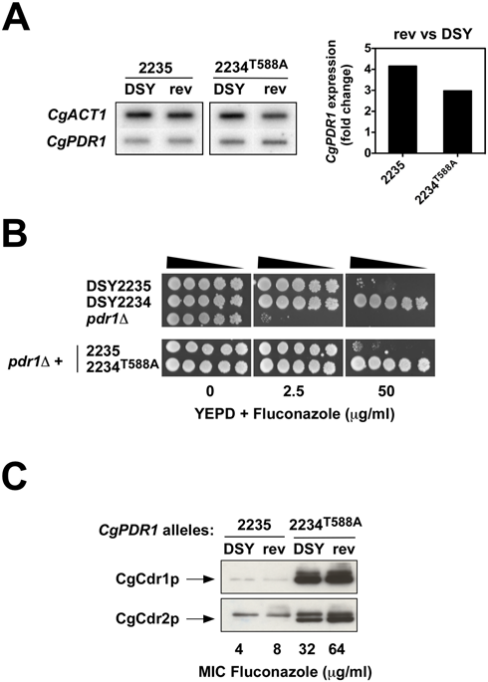
Effect of *CgPDR1* expression on azole resistance. (A) Expression of *CgPDR1* in a matched pair of azole-susceptible and azole-resistant isolates (DSY2235 and DSY2234), in revertant strains (“rev”) overexpressing *CgPDR1* alleles from an episomal plasmid in a *pdr1*Δ mutant derived from DSY562 (SFY53). *CgPDR1* alleles present in each strain (“DSY” for clinical strains and “rev” for revertant strains) were named according to their strain number origin. RNA was isolated from log phase cultures, slot-blotted to membranes, and hybridized with the indicated gene probes. *CgACT1* served as internal control. Signals obtained in blotted membranes were quantified by counting radioactivity by phosphor imaging. Signals obtained for *CgPDR1* were normalized with *CgACT1* and substracted from background. Expression values represent the increase of *CgPDR1* expression in revertant strains relative to the clinical isolates expressing the same *CgPDR1* allele. (B) Fluconazole susceptibility testing of *C. glabrata* clinical isolates DSY2235 and DSY2234, revertant strains (rev) overexpressing *CgPDR1* alleles and of a *pdr1*Δ mutant (SFY53). Isolates were grown on YPD medium containing the drug at the indicated concentration at 30°C for two days. (C) Immunodetection of CgCdr1p and CgCdr2p in *C. glabrata* clinical isolates DSY2235 and DSY2234, in revertant strains (rev) overexpressing *CgPDR1* alleles in a *pdr1*Δ mutant (SFY53). Proteins extract were separated by SDS-10% PAGE and immunoblotted with rabbit polyclonal anti-CgCdr1p and anti-CgCdr2p antibodies as described previously [9]. MICs to fluconazole were determined by broth microdilution method in accordance with the CLSI M27-A2 document (National Committee for Clinical Laboratory Standards, 2002).

To determine whether the two-fold increase in *CgPDR1* expression observed in some isolates was sufficient to induce high levels of CgCdr1p and CgCdr2p and thus azole resistance, *CgPDR1* alleles from an azole-susceptible and an azole-resistant matched isolate (DSY2235 and DSY2234, respectively) were cloned into the CEN-ARS plasmid pCgACU-5 [19] and expressed in a strain lacking *CgPDR1*. The *CgPDR1* alleles from DSY2235 and DSY2234 only differ by the amino acid substitution T588A. Expression of *CgPDR1* alleles from the episomal plasmid resulted in a three- to four-fold increase of *CgPDR1* mRNA in the revertant strains as compared to the clinical isolates (Figure 4A), but no significant change was observed in CgCdr1p and CgCdr2p levels and in azole susceptibility (Figure 4B and 4C). Similar results were obtained by overexpressing other mutated *CgPDR1* alleles (data not shown), indicating that the slight increase in *CgPDR1* expression observed in some resistant isolates could not account for azole resistance.

### *CgPDR1* Mutations Induce Azole Resistance

**Figure 5:**
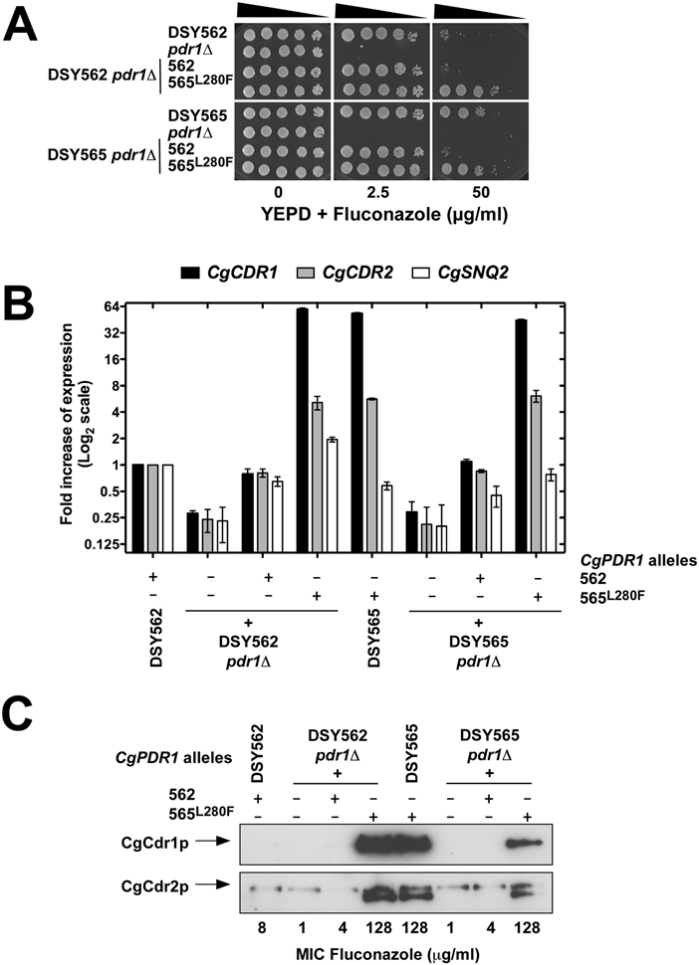
Quantitative analysis of azole resistance in selected *C. glabrata* isolates. (A) Fluconazole susceptibility testing of *C. glabrata* clinical isolates DSY562 and DSY565 and their derivative mutants. Isolates were grown on YPD medium containing the drug at the indicated concentration at 30°C for two days. The indicated genotypes correspond to the following strains: DSY562 *pdr1*Δ: SFY92; DSY565 *pdr1*Δ: SFY94; DSY562 *pdr1*Δ+562 (standing for re-introduction of *CgPDR1* from DSY562): SFY114; DSY562 *pdr1*Δ+565^L280F^ (standing for re-introduction of *CgPDR1* from DSY565 with L280F substitution): SFY115; DSY565*pdr1*Δ+562: SFY118; DSY565 *pdr1*Δ+565^L280F^: SFY119. (B) Expression of *CgCDR1*, *CgCDR2*, and *CgSNQ2* in *C. glabrata* clinical isolates DSY562 and DSY565 and their derivative mutants. Quantification was performed by real-time RT-PCR. The values, which are averages of four separated experiments, represent the increase in gene expression relative to DSY562 (set at 1.00). Error bars show standard deviations. (C) Immunodetection of CgCdr1p and CgCdr2p in *C. glabrata* clinical isolates DSY562 and DSY565 and their derivative mutants. Proteins extract were separated by SDS-10% PAGE and immunoblotted with rabbit polyclonal anti-CgCdr1p and anti-CgCdr2p antibodies as described previously [9]. MICs to fluconazole were determined by broth microdilution method in accordance with the CLSI M27-A2 document.

The identified *CgPDR1* mutations were next investigated for their ability to confer hyperactivity to CgPdr1p by mediating high expression of ABC-transporter genes resulting in azole resistance. For this purpose, *CgPDR1* was first inactivated in a pair of azole-susceptible and azole-resistant isolates (DSY562 and DSY565, respectively) and reintroduced at the *CgPDR1* genomic locus by homologous recombination in the obtained *pdr1*Δ strains. *CgPDR1* alleles from these two isolates only differ by the amino acid substitution L280F in the putative inhibitory domain of CgPdr1p. Disruption of *CgPDR1* in both azole-susceptible and azole-resistant isolates led to a drastic increase of azole susceptibility and to the complete downregulation of both *CgCDR* genes (Figure 5A and 5B), thus confirming the involvement of CgPdr1p in azole resistance. Each DSY562 and DSY565 *pdr1*Δ mutant received the *CgPDR1* wild type or mutated alleles. Expression of *CgCDR1*, *CgCDR2* and *CgSNQ2* in reconstituted strains was restored to similar levels than those of the original clinical isolates (Figure 5B). Expression of the *CgPDR1* allele containing the L280F substitution was sufficient to confer CgCdr1p and CgCdr2p constitutive high expression and thus azole resistance in *C. glabrata* independently on the strain genetic background (Figure 5C).

**Figure 6:**
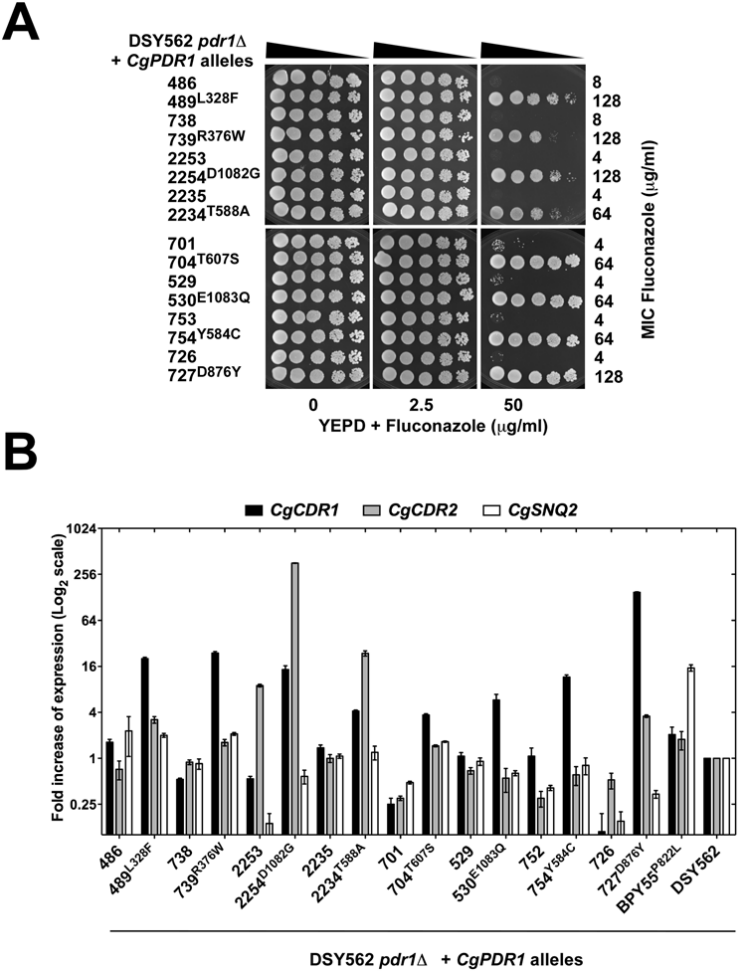
Reconstitution of *CgPDR1* GOF alleles in *C. glabrata*. (A) Fluconazole susceptibility testing of DSY562 *pdr1*Δ mutant strain (SFY93) expressing different *CgPDR1* alleles, which were named according to their strain number origin and by indicating the amino acid substitution (in superscript) associated with a specific strain number. The following strains correspond to the indicated genotypes: DSY562 *pdr1*Δ+486:SFY98; 489^L328F^: SFY99; 738: SFY100; 739^R376W^: SFY101; 2253: SFY102; 2254^D1082G^:SFY103; 2235: SFY104; 2234^T588A^: SFY105; 701: SFY106; 704^T607S^: SFY107; 529:SFY108; 530^E1083Q^: SFY109; 753: SFY110; 754^Y584C^: SFY111; 726: SFY112; 727^D876Y^: SFY113; BPY55^P882L^: SFY116. (B) Expression of *CgCDR1*, *CgCDR2*, and *CgSNQ2* in the DSY562 *pdr1*Δ mutant strain (SFY93) expressing different *CgPDR1* alleles, named according to their strain number origin. Quantification was performed by real-time RT-PCR. The values, which are averages of four separated experiments, represent the increase in gene expression relative to DSY562 (set at 1.00). Error bars show standard deviations.

Selected *CgPDR1* alleles from eight other pairs of isolates (Table 1) were reintroduced at the *CgPDR1* genomic locus in an azole-susceptible background lacking *CgPDR1*. *CgPDR1* alleles from each pair of isolates only differ by a point mutation leading to a single amino acid substitution in either the inhibitory domain, the MHR or the activation domain of CgPdr1p. These mutations, which were specific for azole-resistant isolates, restored azole resistance in a *pdr1*Δ mutant (fluconazole MICs from 64–128 µg/ml, Figure 6A). Since only alleles containing these mutations conferred *CgCDR1* constitutive high expression (from 4- to 150-fold expression increase, Figure 6B), these mutations could be assigned as GOF mutations. Moreover, single amino acid substitutions in either the inhibitory domain, the MHR or the activation domain could confer drug resistance. Once again, altered *CgPDR1* expression could not account for azole resistance, since the *CgPDR1* mRNA levels were similar between the clinical strains and the revertant strains expressing their corresponding *CgPDR1* alleles (Figure S3).

### Distinct *CgPDR1* GOF Mutations Have Differentiated Effects on ABC-Transporter Genes Expression

To determine the effect of distinct mutated *CgPDR1* alleles on ABC-transporter genes expression, mRNA levels of *CgCDR1*, *CgCDR2* and *CgSNQ2* were determined by real-time RT-PCR in 21 matched pairs of azole-susceptible and azole-resistant isolates clinical isolates listed in Table 1 (Figure 1). As previously observed by Torelli *et al.* [17], *CgCDR1*, *CgCDR2* and *CgSNQ2* were not always coordinately expressed in azole-resistant isolates. Among them, *CgSNQ2* showed in general the lowest expression variations. Taking a significant upregulation between two isolates as a threshold of two-fold expression difference, only nine isolates were above this value. *CgSNQ2* was more upregulated (8.5-fold) than the other ABC transporters only in DSY2277 (Figure 1). Upregulation of *CgCDR1* and *CgCDR2* in azole-resistant isolates followed similar patterns but without reaching statistical significance (data not shown). In some cases, significant upregulation (from 13- to 170-fold) of only *CgCDR1* was measured (DSY2268, DSY2273, DSY754, DSY2315, DSY565 and DSY2746). In two cases (DSY2254 and DSY2234), *CgCDR2* upregulation outreached that of *CgCDR1* (Figure 1). Since the three ABC-transporter genes are regulated by *CgPDR1*, their uncoordinated expression might be explained by either differences in their promoter sequences or by differences in the transcriptional capacity of CgPdr1p. To avoid differences due to strain genetic backgrounds, pairs of *CgPDR1* alleles were reintroduced in the same background (DSY562 *pdr1*Δ). mRNA levels of *CgCDR1*, *CgCDR2* and *CgSNQ2* were measured by real-time RT-PCR in the revertant strains (Figure 6B) and compared with those obtained in the clinical isolates (Figure 1). For example, the presence of the Y584C substitution (from *CgPDR1* of DSY754) led to *CgCDR1* upregulation only (17- and 12-fold expression increase, Figure 1 and Figure 6B), whereas the presence of the T588A substitution (from *CgPDR1* of DSY2234) resulted in high mRNA levels of both *CgCDR* genes (4- and 8-fold expression increase for *CgCDR1*, 23- and 30-fold increase for *CgCDR2*, Figure 1 and Figure 6B). Finally the presence of the P822L substitution (from *CgPDR1* of BPY55) in a *pdr1*Δ mutant resulted in the upregulation of *CgSNQ2* only (15-fold, Figure 6B), which is consistent with previous observation made in this clinical isolate [17]. These results as well as those obtained with other engineered isolates were overall consistent with expression profiles from clinical isolates containing the same *CgPDR1* alleles (Figure 1) and thus indicate that ABC-transporter genes might be differentially regulated depending on the *CgPDR1* GOF mutation and independently on the strain background.

### *CgPDR1* GOF Mutations Are Responsible for Increased Virulence of *C. glabrata*

The *C. glabrata* isolates analysed in this study are of clinical origin. These isolates may have adapted to the host conditions in order to cause disease. Moreover, azole therapy in these patients selected for azole-resistant isolates and ultimately led to treatment failure. One interesting and unresolved question is whether *CgPDR1* mutations responsible for high transporter expression have an impact on virulence and fitness of *C. glabrata* under host conditions. We therefore first measured tissue fungal burdens in animal models using tail-vein injections in groups of immuno-competent and immuno-suppressed mice according to previously established protocols [20]–[22]. Fungal loads in kidneys, spleen and liver of all mice were measured seven days after infection. We next performed virulence assays by measuring mice survival after infection with several *C. glabrata* isolates to challenge correlations between virulence and possible differences in tissue fungal loads.

**Figure 7:**
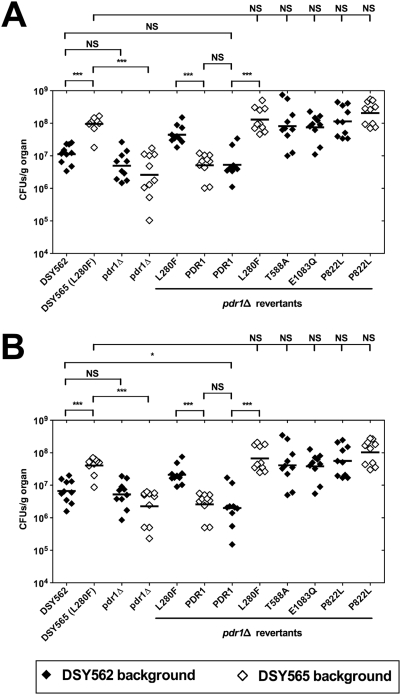
*C. glabrata* tissue burdens in murine infection models. (A) Fungal tissue burdens in kidneys from immuno-competent BALB/c mice infected intravenously with 4×10^7^ viable cells of *C. glabrata* strains. Mice were sacrificed at day 7 post-infection; and results, which are expressed as CFUs per gram of tissue, represent values recorded separately for each of the ten mice. Geometric means are indicated by horizontal bars and asterisks indicate statistically significant differences (*: *P*<0.05; **: *P*<0.01; ***: *P*<0.001). NS indicates no significance (*P*>0.05). The origin of each strain is indicated. Strain background (DSY562 or DSY565) is indicated by filled or empty symbols, respectively. The *pdr1*Δ mutants from strains DSY562 and DSY565 correspond to SFY92 and SFY94, respectively. Revertants constructed from *pdr1*Δ mutants are indicated by the re-introduced GOF mutation or by the re-introduced wild type *CgPDR1* allele from DSY562. Prism 5.0 was used for statistical analysis and data were processed with non-parametric Wilocoxon Rank sum tests. Comparisons are indicated in the Figure (see Table S3 for details) and associate selected data points. (B) Fungal tissue burdens in kidneys from immuno-suppressed mice infected intravenously with 4×10^7^ viable cells of *C. glabrata* strains. BALB/c mice were rendered neutropenic by intraperitoneal administration of cyclophosphamide (200 mg kg^−1^ of body weight per day) three days before challenge and on the day of infection. Mice were sacrificed at day 7 post-infection.

**Figure 8:**
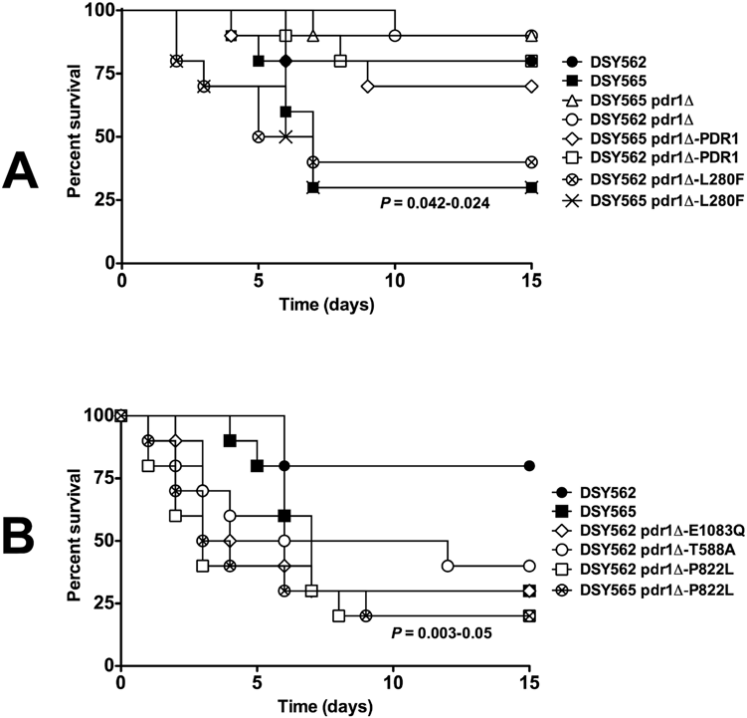
Virulence of *C. glabrata* is dependent on *CgPDR1* GOF mutations. (A) Survival curves of mice infected with DSY562 and DSY565 and derived mutants and revertants. Statistical differences were performed using the Log-rank Mantel-Cox test (Prism 5.0) by comparing survival curves of mice infected by DSY562 and by other strains as indicated. The range of significant *P* values obtained is indicated for DSY565 and strains containing the *CgPDR1* allele with a L280F substitution. (B) Survival curves of mice infected with DSY562 and DSY565 and strains reconstituted with different *CgPDR1* alleles. The range of significant *P* values obtained is indicated for DSY565 and strains containing GOF mutations.

Using first DSY562 and DSY565 in immuno-competent or immuno-suppressed mice (Figure 7A and 7B), our results showed that fungal loads in kidneys were significantly increased in both mouse models infected with DSY565 as compared to DSY562 (*P*<0.0001 and *P* = 0.0007, respectively; see details in Table S3). The same trend was observed in fungal loads of spleen and liver (Figure S4). Higher fungal loads in tissues of mice infected with the azole-resistant isolate DSY565 correlated with a significant increased virulence as compared to DSY562 (Figure 8A). Taken together, these results demonstrate that the azole-resistant isolate (DSY565) was more virulent than its azole-susceptible parent, DSY562. Since DSY562 and DSY565 differ at least by the presence of the L280F substitution in *CgPDR1*, it was tempting to test whether this substitution was responsible for this behavior. We therefore replaced the mutated allele with the *CgPDR1* wild type allele in DSY565 (named as DSY565 pdr1Δ-PDR1; SFY118) and also replaced the *CgPDR1* wild type allele with the mutated allele in DSY562 (named as DSY562 pdr1Δ-L280F; SFY115). *CgPDR1* alleles were also reconstituted in their original backgrounds (DSY562 pdr1Δ-PDR1; SFY114 and DSY565 pdr1Δ-L280F; SFY119). Mice infected with DSY562 pdr1Δ-L280F or DSY565 pdr1Δ-L280F had significant higher fungal loads in their kidneys as compared to DSY565 pdr1Δ-PDR1 or DSY562 pdr1Δ-PDR1 (Figure 7A, *P*<0.0001). The same was observed in immuno-suppressed mice (Figure 7B, *P*<0.0001). No significant changes in kidneys fungal loads occurred between immuno-competent mice infected with DSY562 and DSY562 pdr1Δ-PDR1 (*P* = 0.07) or with DSY562 pdr1Δ-PDR1 and DSY565 pdr1Δ-PDR1 (*P* = 0.62). Virulence assays with the same strains confirmed the association of increased virulence with increased fungal loads of infected tissues (Figure 8A). Thus, increased virulence was associated with the presence of a GOF mutation in *CgPDR1* independently on the strain background. Introducing other mutations in the background of DSY562 mimicked results of fungal tissue burdens and of virulence assays obtained with the first tested *CgPDR1* mutant allele (see results obtained with DSY562 pdr1Δ-T588A, -Q1083Q, -P822L and DSY562 pdr1Δ-P822L, Figure 7 and Figure 8B) and thus one can predict that increased virulence may be caused by any of the GOF mutations so far identified in *CgPDR1*. Interestingly, the absence of *CgPDR1* in DSY562 (DSY562 pdr1Δ; SFY92) did not significantly alter fungal tissue burdens and virulence in animal models (Figure 7 and Figure 8A). This strongly suggests that it is rather the presence of a GOF mutation than the presence of *CgPDR1* that directly affects virulence.

**Figure 9:**
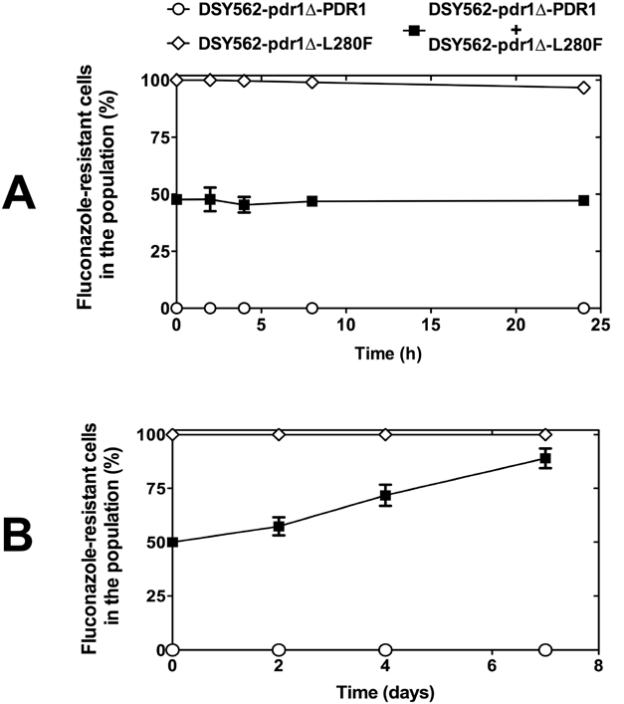
Fitness assays between azole-resistant and azole-susceptible isolates. (A) *In vitro* fitness assays. Strains SFY114 (DSY562 pdr1Δ-PDR1) and SFY115 (DSY562 pdr1Δ-L280F) were inoculated in YEPD in single or mixed (1∶1) cultures. Aliquots were taken from each culture in triplicate and population ratios were calculated from the proportion of azole-susceptible and azole-resistant colonies plated onto YEPD agar as described in Material and Methods. (B) *In vivo* fitness assays. SFY114 (DSY562 pdr1Δ-PDR1) and SFY115 (DSY562 pdr1Δ-L280F) were inoculated intravenously as single or mixed (1∶1) cultures to three groups of mice as described in Material and Methods. Kidneys were taken at given time points from sacrificed mice and population ratios were measured as described above.

This assumption is also based on fitness tests performed *in vitro* and *in vivo* with two selected isolates, each containing a wild type (DSY562 pdr1Δ-PDR1; SFY114) or a mutated *CgPDR1* allele (DSY562 pdr1Δ-L280F; SFY115). We observed that a GOF mutation had no selective advantage *in vitro* for *C. glabrata* over a susceptible parent isolate since both strains cultivated over 24 h at a 1∶1 population ratio remained in equivalent population proportions (Figure 9A). It is only *in vivo* that the azole-resistant population displayed a selective advantage over susceptible isolates. After inoculating mice with a 1∶1 population ratio, a progressive disappearance of the susceptible population was visible over the time lapse of the animal experiment (Figure 9B). These results demonstrate that a GOF in *CgPDR1* is associated with a gain in fitness *in vivo* even in the absence of drug selection.

### *CgPDR1* GOF Mutations Affect the *In Vivo* Response to Fluconazole

**Figure 10:**
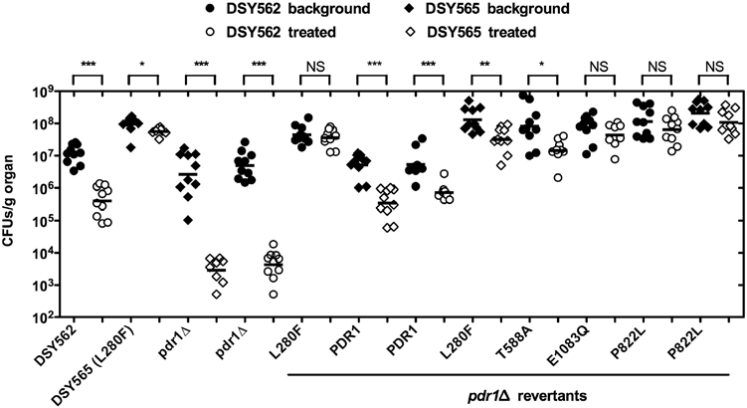
Efficacy of azole treatment in *C. glabrata* assessed by fungal tissue burden in kidneys. Fluconazole (100 mg/Kg/day) was administered by intraperitoneal injection in immuno-suppressed mice as described in Material and Methods. Treatment was initiated 24 h after inoculation (day 1 post-infection) and continued until day 7 post-infection. Mice were injected with 4×10^7^ CFU of each investigated strain and organ homogenates were obtained from ten mice per group that were sacrificed and necropsied on day 8 post-infection. Results, which are expressed as CFUs per gram of tissue, represent means of values recorded separately for each of the mice. Geometric means are indicated by horizontal bars and asterisks indicate statistically significant differences between two conditions (see legend of Figure 7 and Table S3 for details). Closed and empty circles indicate CFU from untreated and fluconazole-treated animals infected with a DSY562 background, respectively. Closed and empty diamonds indicate CFU from untreated and fluconazole-treated isolates with a DSY565 background, respectively.

Since DSY565 is resistant to azoles *in vitro*, we expected little changes between tissue burden of mice infected with this clinical isolate in fluconazole-treated or -untreated conditions. The data obtained after measurement of colony forming units (CFU) from tissues of treated versus untreated animals infected with this strain confirmed this hypothesis only in spleen and liver (Figure S5, *P* = 0.39 and 0.82), while partially in kidneys (Figure 10, *P* = 0.04). These results contrasted with those obtained with the azole-susceptible isolate DSY562: a sharp and significant decrease of fungal load was observed in all organs when comparing treated versus untreated animals (*P*<0.0001–0.03). The absence of *CgPDR1* in both DSY562 and DSY565 backgrounds resulted in this experimental setting in a three-log decrease of fungal loads when animal were treated with fluconazole. This result could be expected from *in vitro* susceptibility data that yielded for these mutants the lowest fluconazole MIC values (Figure 5C). This suggests that *CgPDR1* is essential for the response of *C. glabrata* to fluconazole challenge *in vivo*. Reconstituting wild type isolates from DSY562 and DSY565 backgrounds with a *CgPDR1* wild type allele (DSY562 pdr1Δ-PDR1: SFY114; DSY565 pdr1Δ-PDR1: SFY118) gave in terms of fluconazole efficacy similar results to those obtained with the starting clinical isolate DSY562 (Figure 10). With the exception of DSY562 pdr1Δ-T588A (*P* = 0.02), the reconstitution of GOF mutations in *CgPDR1* in both DSY562 (DSY562 pdr1Δ–Q1083Q, -P822L) and DSY565 (DSY565 pdr1Δ-L280F, -P822L) gave by CFU counts in kidneys no significant differences between fluconazole-treated and untreated animals. However, CFU counts of spleen and liver of animals infected with DSY562 pdr1Δ-T588A were not significantly different from untreated animals upon fluconazole treatment (*P* = 0.28 and 0.16), thus suggesting that this GOF was still responsible for treatment failure. Taken together, our results are in agreement with the idea that high fluconazole MIC values are mirrored by treatment failure in this animal model. Moreover, our results demonstrate the critical role of *CgPDR1* for the adequate response of *C. glabrata* to fluconazole challenge. On the other hand, our results also highlight that *CgPDR1* GOF mutations alone are responsible for fluconazole treatment failure in the experimental model tested here.

## Discussion

CgPdr1p is a Zn(2)-Cys(6) transcription factor involved in the regulation of the ABC-transporter genes *CgCDR1*, *CgCDR2* and *CgSNQ2*, which are responsible for azole resistance in *C. glabrata*. Four single amino acid substitutions in CgPdr1p (W297S, F575L, P822L and P927L) have been reported to increase the expression of at least *CgCDR1*, *CgCDR2* and *CgSNQ2* and thus to contribute to azole resistance of clinical isolates [14],[15]. This work allowed the identification of 57 additional substitutions in CgPdr1p localized in three main “hot spots” near the so-called inhibitory domain, the MHR and the transcriptional activation domain.

### *CgPDR1*: The Expected and the Non-Expected

Nine distinct mutations in *CgPDR1* alleles, three in each of the “hot spots” domains, conferred constitutive upregulation of ABC-transporter genes when expressed in an azole-susceptible background and thus can be considered as true GOF mutations responsible for azole resistance in *C. glabrata*. The 48 other mutations are also likely GOF mutations since they are present only in alleles isolated from azole-resistant strains upregulating ABC-transporter genes and the localization of these mutations along the protein is similar to GOF mutations described in the *S. cerevisiae* homologs Pdr1p/Pdr3p. Most GOF mutations in *PDR3* map to the first two motifs of the inhibitory domain and to the transcriptional activation domain [23]. In contrast, *PDR1* mutations are scattered throughout the entire protein with some “hot spots” at the C-terminus [24]. Given the high diversity of GOF mutations found in *CgPDR1*, it is unlikely that all mutations affect similarly the transcriptional activity of CgPdr1p that is responsible for ABC-transporter genes overexpression. Hence, GOF mutations in the inhibitory domain might impair transcriptional inhibition and those in the transcriptional activation might induce hyperactivation as proposed for *PDR3* mutations in *S. cerevisiae*.

The effect of some of the identified GOF mutations might be enlightened by the recent finding that CgPdr1p acts as nuclear receptor by directly binding to azoles in order to activate expression of efflux pumps genes [16]. For instance, the xenobiotic binding domain of *S. cerevisiae* Pdr1p has been mapped between amino acids 352 and 543, and this domain corresponds by sequence alignment to the MHR region of CgPdr1p. GOF mutations in the MHR might bypass the requirement of xenobiotic binding that is otherwise necessary to activate the transcription of efflux pumps genes. Determining whether *CgPDR1* alleles containing mutations in the MHR domain could still be induced by azoles may verify this hypothesis. Once bound to azoles, CgPdr1p forms a complex with the Mediator co-activator subunit CgGal11p though the KIX motif to activate transcription of target genes. The CgPdr1p KIX-binding domain has been mapped between amino acids 1074 to 1104 [16]. Interestingly, we identified nine different GOF mutations within this short motif, suggesting that GOF mutations might modify CgGal11p recruitment in absence of drug binding.

It is now well established that *S. cerevisiae* Pdr1p and Pdr3p act through *cis* acting sites present in the promoters of target genes. The consensus motif is named PDRE (for pleiotropic drug resistance element) and is present in several ABC-transporter gene promoters such as *PDR5*, *SNQ2* and *YOR1* [25]. In *C. glabrata*, a genome-wide study identified genes upregulated by a *CgPDR1* GOF mutant and, by analysis of the promoters, the sequence 5′-TCC(GA)(CT)GAA-3′ was identified as a strong candidate for *C. glabrata* PDRE. This sequence is found in the promoters of *CgCDR1*, *CgCDR2* and *CgSNQ2*, suggesting that CgPdr1p binds directly to PDRE sequences to regulate transcription of target genes [14],[15],[17]. Surprisingly, our results show that *CgCDR1*, *CgCDR2* and *CgSNQ2* are not coordinately expressed in azole-resistant isolates. Moreover, the selective upregulation of efflux pump genes is dependent on the *CgPDR1* GOF alleles, since expression of distinct mutated alleles in the same genetic background restored the ABC-transporter gene expression of the parental clinical strains from which the alleles were isolated. This observation highlights still underscored and novel functions of *CgPDR1* on the regulation of target genes. One possible explanation for these differentiated effects could be the localization of the mutations that may alter the transcriptional activity of CgPdr1p. However, data presented here indicate that mutations within the same domain do not yield similar ABC-transporter expression patterns. For example, two distinct mutations in the KIX domain, E1083Q (in DSY530) and D1082G (in DSY2254), gave selective upregulation of *CgCDR1* alone or both *CgCDR1* and *CgCDR2*, respectively. On the other hand, differences in the number and/or sequence of the PDRE present in the efflux pump promoters could influence CgPdr1p regulatory activity. Alternatively, the function of CgPdr1p might be subjected to regulation by other transcription factors. Irrespective of the precise molecular mechanism involved, our results strongly suggest that *CgPDR1* GOF mutations have differentiated effects on target genes including the major ABC-transporters involved in azole resistance. Microarray experiments performed with individual *CgPDR1* mutations would help determining whether other target genes are differentially expressed and promoter analysis may provide clues to uncover this mechanism.

The presence of a PDRE sequence in the promoter of *CgPDR1* as well as the constitutive high expression of *CgPDR1* in some azole-resistant isolates suggested an auto-regulation similarly to *PDR3* [14],[15],[26]. Moreover, enhanced *CgPDR1* expression was also observed in some petite mutants [15]. It has thus been proposed that *CgPDR1* may regulate its own expression leading to azole resistance. However, results presented here suggest that *CgPDR1* upregulation is restricted to a limited number of azole-resistant isolates and does not correlate with the presence of GOF mutations. The increase in *CgPDR1* expression observed in our strains is not sufficient *per se* to induce azole resistance. This observation is consistent with a recent study demonstrating that *CgPDR1* expression is poorly correlated with azole resistance in *C. glabrata* [27]. Even though our experimental approaches were different from published studies, especially with respect to the engineering of mutant and revertant strains, the regulation of *CgPDR1* needs to be further investigated.

### CgPDR1 and Effects on *C. glabrata*-Host Interactions

During the last decades, there has been a significant increase in the appearance of resistance in *C. glabrata* as a result of an increased use of azoles combined with the exceptional ability of this yeast species to develop resistance. In bacteria, antimicrobial resistance is often associated with fitness costs and thus results in a competitive disadvantage against otherwise drug-susceptible bacteria within the host [28]. Restoration of fitness in drug-resistant bacteria is often associated with the emergence of compensatory mutations [29]. A few examples illustrate that mutations in genes involved in antibiotic resistance can be beneficial in the fitness of pathogenic bacteria [30],[31]. Implicit in this reasoning is that antifungal resistance may have fitness costs in fungi and thus may result in a counter-selection against resistant strains without drug pressure. Whether resistance similarly exerts a relevant fitness cost that is associated with diminished virulence in fungi is still debated [28]. One study has addressed this question in *C. albicans* by testing the virulence of azole-resistant isolates compared to their azole-susceptible parental isolate [32]. The authors concluded that no direct relationship exists between the development of azole resistance and virulence. *In vitro* studies showed that individual *C. albicans* colonies subcultured in fluconazole-containing medium can follow individual emergence of azole resistance mechanisms. Some of these trajectories can be associated with an immediate decrease in fitness (measured by reduced growth rate). However fitness is restored by further cultivation by still unknown compensatory mechanisms [33]. Other studies suggest that once the selective pressure eases, the fungal-resistant strains will disappear [34]–[36].

Testing the interplay between *C. glabrata* and the host requires a validated animal model. Existing studies have mostly used intravenous injection of *C. glabrata* at varying doses (10^7^ to 10^8^ cells per mouse) with different immune system status (immuno-competent and immuno-suppressed) and in different mice backgrounds [37],[38]. Depending on the initial infection doses and the mice genetic background, not only CFU counts in target organs (kidneys, spleen and liver) differed by several logs (from 10^5^ to 10^8^ CFU per g infected tissue) over the duration of the experiments but also mice survival was varying. As compared to *C. albicans*, *C. glabrata* was consistently much less pathogenic. In our study, we have followed protocols established recently [17],[21] and used different immune status regimen to enable valid conclusions on the relationship between azole resistance and virulence. Our findings that *C. glabrata* resistant strains are both more virulent in mice and less susceptible to azoles *in vitro*/*in vivo* as compared to wild type isolates strongly suggest a gain in fitness for the resistant isolates. As shown in this study (Figure 9B), the azole-resistant population did effectively take over the azole-susceptible isolates in the absence of drug selection. We showed that it is the presence of GOF mutations rather than the presence of *CgPDR1* that accounts for increased virulence. On the opposite to known *C. glabrata* mutants (such as *ace2*Δ) for which hypervirulence was associated with formation of pseudo-hyphae [39], the morphology of azole-resistant isolates tested here was not altered and therefore GOF mutations in *CgPDR1* are likely to account for the observed phenotype. Hypervirulence has been also observed in other fungal species such as *Cryptococcus neoformans* where perturbation in cAMP signalling by inactivation of *PKR1* (encoding the PKA regulatory subunit) resulted in capsule overproduction [40]. Even though other *C. glabrata* infection models remain to be tested, this is to our knowledge the first example of *in vivo* acquired mutations in a fungal gene with a positive impact on *in vivo* fitness. This highlights a need of carefully monitoring drug resistance of *C. glabrata* in infected patients, since increased virulence observed here may also have a negative impact on the outcome of the disease. In *C. glabrata*, the gain in fitness may have favoured an important proportion of azole-resistant *C. glabrata* isolates with ABC transporter upregulation [4],[13]. This is in contrast with studies on molecular epidemiology of drug resistance performed with other fungal pathogens, where resistance develops by more diverse resistance mechanisms, thus raising the question whether the gain of fitness mediated by *CgPDR1* is a feature unique for *C. glabrata*. It will be therefore interesting to test whether transcriptional activators of drug resistance genes have similar effects on virulence and fitness in other important fungal pathogens.

How *CgPDR1* GOF mutations may affect virulence is still unknown. Nevertheless, published microarray experiments comparing a wild type *C. glabrata* isolates with a resistant strain expressing a mutated form of CgPdr1p may help identifying genes having a putative role in the virulence on the basis of differentially expressed genes [14]. Even though the number of differentially regulated genes is high in this study, several genes involved in stress response, resistance to DNA damage and cell wall structure are upregulated in the azole-resistant isolate, all of which may contribute individually or in combination to modulate the virulence of *C. glabrata*. Further studies are therefore necessary to address the involvement of these genes in virulence.

Our results demonstrate that *CgPDR1* GOF mutations alone are responsible for fluconazole treatment failure in a murine model. In most cases, animals infected with isolates containing GOF mutations were not responding to fluconazole treatment on the basis of CFU counts in specific organs (Figure 10 and Figure S5). Fluconazole is not considered as a therapeutic option for *C. glabrata* infections in human but served here as a compound to test the effect of GOF mutations on treatment outcome. Our results showed that a *C. glabrata* isolate with a low azole MIC can still respond to fluconazole treatment. A reduction of *C. glabrata* CFU counts in the range observed in the present study in azole-treated animals (approximately 1.5 logs) is not consistently reported in experimental *C. glabrata* candidiasis [41]–[44]. These variations probably reflect the technical difficulties behind the establishment of a disease by *C. glabrata* in different animal models but could also be the result of inappropriate drug regimen. Having determined conditions necessary to establish a *C. glabrata* infection and to respond to drug treatment by a corresponding drug dosage, our study could demonstrate that *in vitro* fluconazole resistance was well correlated with *in vivo* resistance. Because GOF mutations in *CgPDR1* are also responsible for increased virulence, drug treatment faces the challenges of both higher fungal loads and acquired resistance. Under these conditions, the failure of drug treatment is more likely. Besides the role of *CgPDR1* GOF mutations in azole treatment failure, *CgPDR1* was shown to play a critical role for the response of *C. glabrata* during fluconazole treatment as observed by the sharp decrease in fungal tissue burden after treatment in animals infected with mutants lacking *CgPDR1*. Together with the recent discovery that *CgPDR1* possesses functional domains used as drug ligands and therefore potential sites for inhibitors [16],[45], our data suggest that inhibition of *CgPDR1* could shortcut fitness and potentiate azole therapy. Future studies are therefore necessary to explore this possibility.

## Materials and Methods

### Strains and Growth Media

The *C. glabrata* strains used in this study are listed in Table S1. One hundred twenty two clinical isolates of *C. glabrata* and were recovered from different specimens (e.g. blood, urine, vagina, sputum) of patients. Strains with the prefix “BPY” were collected at the Università Cattolica del Sacro Cuore, Rome, Italy. Strains with the prefix “DSY” were collected at the University Hospital Center, Lausanne (Switzerland) except strains DSY1166, DSY1169, DSY1174, DSY1176, DSY1180, DSY1185 that were provided by from Nippon Roche Research (Kanagawa, Japan). Strains DSY2724, DSY2725, DSY2726, DSY2731, DSY2746 were obtained from J.-P. Bouchara at the Centre Hospitalier d'Angers (France) and strains DSY2769, DSY2770 from M.-E. Bougnoux at the Necker Hospital (Paris, France). Strains were stored in 20% glycerol stocks at −80°C and cultured on either YPD (1% yeast extract, 2% peptone, 2% glucose) or minimal medium YNB (0.67% yeast nitrogen base plus 2% glucose) at 30°C when necessary. For solid media, 2% agar was added. YNB with appropriate amino acids and bases was used as a selective medium after transformation of yeast strains [10]. YEPG (1% yeast extract, 2% peptone, 3% glycerol, 1% ethanol) agar plates was used to test *C. glabrata* strains for petite growth phenotype. YPD agar plates containing nourseothricin (clonNAT, Werner BioAgents) at 200 µg ml^−1^ were used as a selective medium for growth of yeast transformant strains. Drugs were obtained from the following sources: fluconazole (Pfizer), itraconazole and ketoconazole (Janssen). *Escherichia coli* DH5α was used as a host for plasmid construction and propagation. DH5α was grown in Luria-Bertani broth or on Luria-Bertani agar plates supplemented with ampicillin (0.1 mg ml^−1^) when required.

### Drug Susceptibility Assays

The *C. glabrata* strains were tested for azole susceptibility with the broth microdilution method described in the CLSI (formerly NCCLS) document M27-A2 (National Committee for Clinical Laboratory Standards, 2002). Briefly, aliquots of 1.5 (±1.0)×10^3^ cells ml^−1^ were distributed to wells of a microtitre plate in RPMI 1640 containing 2% glucose and incubated at 35°C for 48 h. Endpoint readings were recorded with an automatic plate reader (Multiskan Ascent, Thermo) and the lowest azole concentration that reduced growth to 50% of that of the drug-free control was defined as the MIC. Susceptibility to fluconazole of *C. glabrata* strains was tested qualitatively by spotting serial dilutions of overnight-grown yeast broth cultures onto YPD agar plates with different drug concentrations, as described previously [10]. After incubation at 30°C for 48 h, yeast spots were visualized onto plate surfaces.

### Sequencing of *CgPDR1*

*C. glabrata* genomic DNA was used as a template to amplify by PCR *CgPDR1* using the primers CgPDR1-EcoRI (5′-ATACCAGAATTCGGTCTCCGCTACAGGTTATA-3′) and CgPDR1-BamHI (5′-AAGTTTGGATCCAACGTTGTTGAGAAGGTATT-3′). The resulting PCR product was sequenced with the BigDye Terminator v1.1 Cycle Sequencing Kit (Applied Biosystems) according to the manufacturer protocol.

### Immunoblotting

*C. glabrata* cell extracts for immunoblotting were prepared by an alkaline extraction procedure as described previously [46]. Detection of CgCdr1p and CgCdr2p was performed as described previously [9]. Signals were revealed by exposure to Amersham Hyperfilm MP films (GE Healthcare).

### Quantitative Real-Time RT-PCR

Total RNA was extracted from log phase cultures with an RNeasy Protect Mini kit (Qiagen) by a process involving mechanical disruption of the cells with glass beads and an RNase-free DNase treatment step as previously described [13]. Expression of the *CgCDR1*, *CgCDR2* and *CgSNQ2* genes was quantitatively assessed with real-time RT-PCR in an i-Cycler iQ system (Bio-Rad). All primers and probes [13] were designed with Beacon Designer 2 (version 2.06) software (Premier Biosoft International) and synthesized by MWG Biotech (Ebersberg, Germany). RT-PCRs were carried out as previously described [13]. Each reaction was run in triplicate on three separate occasions. For relative quantification of the target genes, each set of primer pairs and the Taqman probes were used in combination with the primers and probe specific for the *CgACT1* reference gene in separate reactions [17]. Changes (*n*-fold) in gene expression relative to that of DSY562 (azole-susceptible control isolate) were determined from *CgACT1*-normalized expression levels. A two-fold increase in the expression level of each gene was arbitrarily considered as significant [17]. Expression of *CgPDR1* in related azole-susceptible and azole-resistant clinical isolates was determined by real-time RT-PCR in an ABI Prism 7000 (Applied Biosystems). Each reaction was run in triplicate on three separate occasions. *CgPDR1* quantification was performed using the primers CgPDR1-for (5′-AGCCTTGCCGATAGTCATAC-3′) and CgPDR1-rev (5′-AAGGTCAGGGCATACTTCAG-3′) using the QuantiTect SYBR Green PCR Kit (Qiagen). CgPDR1 alleles sequenced in this work can be found under GenBank accession numbers FJ550215 to FJ550284. *CgPDR1* expression was normalized by *CgACT1* expression levels using the specific primers CgACT1-for (5′-TTCCAGCCTTCTACGTTTCC-3′) and CgACT1-rev (5′-TCTACCAGCAAGGTCGATTC-3′).

### RNA Hybridization

Small-scale isolation of RNA from *C. glabrata* was performed as previously described [10]. Five µg of denaturated RNA was transferred under vacuum onto GeneScreen Plus membranes (Perkin Elmer) using the slot-blotter MINIFOLD II (Schleicher & Schuell). Membranes were washed in 2× SSC and dried during 2 h at 80°C under vacuum. Probes were labelled by random priming with [α-^32^P]dATP using the Mega Labeling kit (GE Healthcare) according to the instructions of the manufacturer. Radioactive signals were revealed by exposure to Amersham Hyperfilm MP films (GE Healthcare). Signals obtained in blotted membranes were quantified by counting of radioactivity with the help of a Typhoon Trio (GE Healthcare). The *CgPDR1* probe corresponds to the entire open reading frame and was generated by PCR using primers CgPDR1-SphI (5′-GCGCAAAGCATGCATG CAAACATTAGAAACTACA-3′) and CgPDR1-10 (5′-TCCTTAAGCCCGATAAGG-3′). The *CgACT1* probe was used as an internal standard and was generated by PCR using primers CgACT1F (5′-TTGACAACGGTTCCGGTATG-3′) and CgACT1R (5′- CCGCATTCCG TAGTTCTAAG-3′).

### Expression of *CgPDR1* Alleles in SFY53

To express *CgPDR1* alleles in an azole-susceptible strain, the *CgPDR1* ORF flanked by 1.2 kb-upstream and 0.5 kb-downstream regions was amplified by PCR from DSY2235 and DSY2234 genomic DNA using the primers CgPDR1-EcoRI and CgPDR1-BamHI (see above) and inserted into pCgACU-5 to yield pSF18 and pSF19, respectively. These plasmids were used to transform SFY53 (DSY562 pdr1Δ) to obtain SFY72 and SFY73, respectively.

### Disruption of *CgPDR1*

pSF2, in which the *SAT1* flipper is flanked by *CgPDR1* upstream and downstream sequences, was used for the disruption of *CgPDR1* in DSY562 and DSY565 [17]. This plasmid was linearized by digestion with *Kpn*I and *Sac*I and transformed into DSY562 and DSY565 to obtain after selection of transformants on nourseothricin-containing YPD plates the *CgPDR1* deletion strains SFY92 and SFY93, respectively.

### Replacement of *CgPDR1* Alleles

For *CgPDR1* replacement in SFY92 and SFY94, FLP-mediated excision of the *SAT1* cassette was first induced by growing the cells for 4 h at 30°C in YCB-BSA medium (23.4 g l^−1^ yeast carbon base and 4 g l^−1^ bovine serum albumin; pH 4.0). One hundred to 200 colonies were plated onto YPD plates containing nourseothricin (15 µg ml^−1^) and grown for 48 h at 30°C to obtain nourseothricin-sensitive strains SFY93 and SFY95, respectively.

The revertant strains generated in this study were obtained by transformation of the *pdr1*Δ mutants SFY93 and SFY95 with linearized plasmids containing the *SAT1* marker and the PCR-amplified *CgPDR1* open reading frames of the strains listed in Table 1 as described previously [17]. Briefly, the complete *CgPDR1* ORF flanked by 500 bp was amplified by PCR from genomic DNA of the first eight strain pairs of Table 1 using primers CgPDR1-KpnI (5′-GCAAAGGTACCCGTTGATCATTATAATTGTGGGTAAA-3′) and 3′UTR-PDR1-SacI (5′-GCGCAAAGAGCTCGAGTTACAGACGACCAACGTGTCG-3′) and inserted into pBluescript II SK(+). These plasmids were amplified by PCR using the primers CgPDR1-EcoRI inv (5′-GCGCAAAGAATTCGTTGAGAAGGTATTAAGAACTTC-3′) and 3′UTR-PDR1-NotI (5′-GCGCAAAGCGGCCGCTACCGAAAGTTCGGTAAATCTAGG-3′). The resulting PCR products were digested by *Eco*RI and *Not*I and ligated to a 1.8 kb *Eco*RI/*Not*I fragment containing *SAT1* from pSFS1. The resulting plasmids were linearized by *Kpn*I and *Sac*I and transformed into SFY93 and SFY95 to obtain, after selection of transformants on nourseothricin-containing YPD plates, the *CgPDR1* revertant strains listed in Table S1.

### Animal Studies

Female BALB/c mice (20 to 25 g; Harlan Italy S.r.l) were housed in filter-top cages with free access to food and water, and were used for all in vivo experiments with the approval by the institutional Animal Use Committee (Università Cattolica del Sacro Cuore, Rome Roma, Italy). To establish *C. glabrata* infection, mice were injected into their lateral vein with saline suspensions of the *C. glabrata* strains (each in a volume of 200 µl).

In virulence studies, a group of ten mice was established for each yeast strain. In a first experiment series, immuno-competent or immuno-suppressed mice were inoculated with 4×10^7^ colony-forming units (CFU) of the yeasts [21]. Mice were rendered neutropenic by intraperitoneal administration of cyclophosphamide (200 mg kg^−1^ of body weight per day) three days before challenge and on the day of infection. After seven days, mice were sacrificed by use of CO_2_ inhalation, and target organs (liver, spleen and kidney) were excised aseptically, weighted individually and homogenized in sterile saline by using a Stomacher 80 device (Pbi International) for 120 s at high speed. Organ homogenates were diluted and plated onto YPD. Colonies were counted after two days of incubation at 30°C, and the numbers of CFU g^−1^ of organ were calculated. In a second experiment, mice were rendered neutropenic as above described and were injected with 7×10^7^ CFUs of the strains [22]. Mice were monitored with twice-daily inspections and those that appeared moribund or in pain were sacrificed by use of CO_2_ inhalation.

In fluconazole treatment studies, two groups of tenmice, one for drug treatment and one for control, were established with each strain. Neutropenia was induced as above described on days −4, +1 and +4 post-infection [20]. Mice were injected with 4×10^7^ CFUs [20] of the strains and were sacrificed one day after the end of therapy to assess organ fungal burden (see above). Mice received daily intraperitoneal injections of 100 mg kg^−1^ fluconazole diluted in saline [41] and the treatment was initiated 24 h after challenge and continued through post-infection day 7.

CFU counts were analysed with non-parametric Wilocoxon Rank sum tests, while mean survival times were compared among groups by using the long-rank test. A *P*-value of less than 0.05 was considered to be significant. All relevant *P*- values calculated in this study are listed in Table S3.

### Measurement of SFY114 and SFY115 Fitness *In Vitro* And *In Vivo*

Strains SFY114 and SFY115 were grown overnight in YEPD and diluted to a density of 5×10^6^ cells ml^−1^. Equal volumes of each culture were mixed together and cultures were grown under constant agitation at 30°C for 24 h. Growth of SFY114, SFY115 and the co-culture was determined at 2 h, 4 h, 8 h and 24 h by measuring the absorbance at 540 nm and by plating diluted samples of the cultures onto YEPD agar plates. Since SFY115 is able to grow on high concentrations of fluconazole in contrast to SFY114, the relative proportion of both strains in the co-culture was determined by replicating colonies onto YEPD agar containing 30 µg^−1^ ml of fluconazole. After incubation at 30°C for 48 h, colonies on YEPD plates and on plates containing fluconazole were counted.

For *in vivo* fitness assays, cultures of strains SFY114 and SFY115 were diluted to a density of 4×10^7^ CFUs and these suspensions were used to infect three groups of mice (four per group). Two groups of mice were infected with SFY114 and SFY115, respectively, and the third group with both strains mixed at a ration of 1∶1. At two, four and seven days post-infection, mice were sacrificed and kidneys homogenates were obtained (see above). Diluted samples from these homogenates were plated onto YEPD. Colonies grown after two days of incubation at 30°C were replicated onto YEPD plates containing fluconazole (30 µg^−1^ ml) to determine the relative proportion of both strains as above-described.

## Supporting Information

Figure S1 Immunodetection of CgCdr1p and CgCdr2p in *C. glabrata*. Panel A: Sequential and related isolates. Panel B: Unrelated clinical isolates. Proteins extract were separated by SDS-10% PAGE and immunoblotted with rabbit polyclonal anti-CgCdr1p and anti-CgCdr2p antibodies as described previously [10]. MICs to fluconazole were determined by broth microdilution method in accordance with the CLSI M27-A2 document (National Committee for Clinical Laboratory Standards, 2002).
(3.03 MB TIF)

Figure S2 Mitochondrial dysfunction in clinical isolates. Panel A: Ability of *C. glabrata* clinical isolates to grow on medium containing glucose (YEPD) or glycerol (YEPG) as carbon source. Azole-susceptible strains DSY2281, DSY2324 and BPY41 are respiratory competent while their matched azole-resistant isolates, DSY2282, DSY2325 and BPY41, respectively, show mitochondrial dysfunction. Panel B: Staining of *C. glabrata* mitochondrial DNA with the fluorescent dye SYTO18 and examined by either phase-contrast (Nomarsky) or fluorescence microscopy. Mitochondrial DNA was stained using SYTO18 (Molecular Probes). *C. glabrata* cells growing exponentially in YEPD at 30°C were harvested, suspended in 10 mM HEPES buffer pH 7.4 with 5% glucose, at 10^6^ cells ml^−1^ and mixed with SYTO18 to a final concentration of 10 mM to stain at 25°C for 5 min. The stained cells were washed and suspended with 10 mM HEPES and observed under a fluorescence microscope.
(1.47 MB TIF)

Figure S3 Expression of *CgPDR1* in matched pairs of clinical isolates (DSY), in revertant strains (rev) and in a *pdr1*Δ mutant (SFY92). RNA was isolated from log phase cultures, slot-blotted to membranes and hybridized with the indicated gene probes. *CgACT1* served as internal control. Signals obtained in blotted membranes were quantified by counting radioactivity with phosphor imaging. Signals obtained for *CgPDR1* were normalized with *CgACT1*. Expression values represent the increase of *CgPDR1* expression in revertant strains relative to the clinical isolates expressing the same *CgPDR1* allele. Since slot-blot quantification of *CgPDR1* expression was comparable to real time RT-PCR (see Fig. 3), only slot-blot data are shown. *CgPDR1* alleles present in each strain (“DSY” for clinical strains and “rev” for revertant strains) are named according to their strain numbers and to their associated *CgPDR1* amino acid substitution (in superscript).
(1.94 MB TIF)

Figure S4 Virulence assays in murine infection models. Panel A: Fungal tissue burdens in spleen from immuno-competent BALB/c mice infected intravenously with 4×10^7^ viable cells of *C. glabrata* strains. Mice were sacrificed at day 7 post-infection and results, which are expressed as CFUs per gram of tissue, represent means of values recorded separately for each of the ten mice. Geometric means are indicated by horizontal bars and asterisks indicate statistically significant differences (*: P<0.05; **: P<0.01; ***: P<0.001). NS indicates no significance (P>0.05). Prism 5.0 was used for statistical analysis and data were processed with non-parametric Wilocoxon Rank sum tests. Comparisons are indicated in the Figure (see Table S3 for details) and associate selected data points. The origin of each strain is indicated. Strain background (DSY562 or DSY565) is indicated by filled or empty symbols, respectively. The *pdr1*Δ mutants from strains DSY562 and DSY565 correspond to SFY92 and SFY94, respectively. Revertants constructed from *pdr1*Δ mutants are indicated by the re-introduced gain of function mutation or by the re-introduced wild type *CgPDR1* allele from DSY562. Panel B: Fungal tissue burdens in spleen from immuno-suppressed mice infected intravenously with 4×10^7^ viable cells of *C. glabrata* strains. BALB/c mice were rendered neutropenic by intraperitoneal administration of cyclophosphamide (200 mg kg^−1^ of body weight per day) three days before challenge and on the day of infection. Mice were sacrificed at day 7 post-infection. Panel C: Fungal tissue burdens in liver from immuno-competent mice. Panel D: Fungal tissue burdens in liver from immuno-suppressed mice.
(0.71 MB TIF)

Figure S5 Efficacy of azole treatment in *C. glabrata*. Panel A: Fungal tissue burdens in spleen from untreated (filled symbols) and fluconazole-treated (open symbols) animals. Panel B: Fungal tissue burdens in liver from untreated (filled symbols) and fluconazole-treated (open symbols) animals. Fluconazole (100 mg/Kg/day) was administered by intra-peritoneal injection in immuno-suppressed mice as described in Material and Methods. Treatment was initiated 24 h after inoculation (day 1 post-infection) and continued through day 7 post-infection. Mice were injected with 4×10^7^ CFU of each investigated strain and organ homogenates were obtained from ten mice per group that were sacrificed and necropsied on day 8 post-infection. Results, which are expressed as CFUs per gram of tissue, represent means of values recorded separately for each of the ten mice. Geometric means are indicated by horizontal bars and asterisks indicate statistically significant differences between two conditions (*: P<0.05; **: P<0.01; ***: P<0.001). NS indicates no significance (P>0.05). Prism 5.0 was used for statistical analysis and data were processed with non-parametric Wilocoxon Rank sum tests (see Table S3 for details).
(0.63 MB TIF)

Table S1 Strains used in this study.
(0.34 MB DOC)

Table S2 Position of mutations of all *CgPDR1* alleles.
(0.15 MB XLS)

Table S3 Details of statistical analysis.
(0.27 MB DOC)
