# Supplementary material for: Gain of Function Mutations in CgPDR1 of Candida glabrata Not Only Mediate Antifungal Resistance but Also Enhance Virulence
Source: PLoS Pathog. 2009 Jan 16;5(1):e1000268. doi: 10.1371/journal.ppat.1000268 (PMC2607542; doi:10.1371/journal.ppat.1000268)
Supplement: Table S1 — Strains used in this study. (0.34 MB DOC) [file ppat.1000268.s006.doc]

**TABLE S1:** Strains used in this study

| **Strain** | **Parental strain** | **Genotype** | **Reference** |
| --- | --- | --- | --- |
| Related clinical isolates | | | |
| DSY486 | Related to DSY489 | Azole-susceptible clinical strain | This study |
| DSY489 |  | Azole-resistant clinical strain | This study |
| DSY738 | Related to DSY739 | Azole-susceptible clinical strain | This study |
| DSY739 |  | Azole-resistant clinical strain | This study |
| DSY2253 | Related to DSY2254 | Azole-susceptible clinical strain | This study |
| DSY2254 |  | Azole-resistant clinical strain | This study |
| DSY2235 | Related to DSY2234 | Azole-susceptible clinical strain | This study |
| DSY2234 |  | Azole-resistant clinical strain | This study |
| DSY701 | Related to DSY704 | Azole-susceptible clinical strain | This study |
| DSY704 |  | Azole-resistant clinical strain | This study |
| DSY2256 | Related to DSY2257 | Azole-susceptible clinical strain | This study |
| DSY2257 |  | Azole-resistant clinical strain | This study |
| DSY759 | Related to DSY2268 | Azole-susceptible clinical strain | This study |
| DSY2268 |  | Azole-resistant clinical strain | This study |
| DSY2270 | Related to DSY2271 | Azole-susceptible clinical strain | [1] |
| DSY2271 |  | Azole-resistant clinical strain | [1] |
| DSY2272 | Related to DSY2273 | Azole-susceptible clinical strain | This study |
| DSY2273 |  | Azole-resistant clinical strain | This study |
| DSY2276 | Related to DSY2277 | Azole-susceptible clinical strain | This study |
| DSY2277 |  | Azole-resistant clinical strain | This study |
| DSY2278 | Related to DSY2279 | Azole-susceptible clinical strain | This study |
| DSY2279 |  | Azole-resistant clinical strain | This study |
| DSY2281 | Related to DSY2282 | Azole-susceptible clinical strain | This study |
| DSY2282 |  | Azole-resistant clinical strain | This study |
| DSY529 | Related to DSY530 | Azole-susceptible clinical strain | [1] |
| DSY530 |  | Azole-resistant clinical strain | [1] |
| DSY753 | Related to DSY754 | Azole-susceptible clinical strain | This study |
| DSY754 |  | Azole-resistant clinical strain | This study |
| DSY755 | Related to DSY756 | Azole-susceptible clinical strain | [1] |
| DSY756 |  | Azole-resistant clinical strain | [1] |
| DSY2316 | Related to DSY2315 | Azole-susceptible clinical strain | This study |
| DSY2315 |  | Azole-resistant clinical strain | This study |
| DSY773 | Related to DSY774 | Azole-susceptible clinical strain | This study |
| DSY774 |  | Azole-resistant clinical strain | This study |
| DSY2317 | Related to DSY717 | Azole-susceptible clinical strain | This study |
| DSY717 |  | Azole-resistant clinical strain | This study |
| DSY726 | Related to DSY727 | Azole-susceptible clinical strain | This study |
| DSY727 |  | Azole-resistant clinical strain | This study |
| DSY2324 | Related to DSY2325 | Azole-susceptible clinical strain | [1] |
| DSY2325 |  | Azole-resistant clinical strain | [1] |
| DSY562 | Related to DSY565 | Azole-susceptible clinical strain | [2] |
| DSY565 |  | Azole-resistant clinical strain | [2] |
| DSY1166 | Related to DSY1169 and DSY1174 | Azole-susceptible clinical strain | This study |
| DSY1169 |  | Azole-resistant clinical strain | This study |
| DSY1174 |  | Azole-resistant clinical strain | This study |
| DSY1176 | Related to DSY1180 and DSY1185 | Azole-susceptible clinical strain | This study |
| DSY1180 |  | Azole-resistant clinical strain | This study |
| DSY1185 |  | Azole-resistant clinical strain | This study |
| DSY2724 | Related to DSY2725 and DSY2726 | Azole-susceptible clinical strain | This study |
| DSY2725 |  | Azole-resistant clinical strain | This study |
| DSY2726 |  | Azole-resistant clinical strain | This study |
| DSY2731 | Related to DSY2746 | Azole-resistant clinical strain | This study |
| DSY2746 |  | Azole-resistant clinical strain | This study |
| BPY40 | Related to BPY41 | Azole-susceptible clinical strain | [3] |
| BPY41 |  | Azole-resistant clinical strain | [3] |
| BPY112 | Related to BPY126 | Azole-susceptible clinical strain | [3] |
| BPY126 |  | Azole-resistant clinical strain | [3] |
| BPY241 | Related to BPY285 | Azole-susceptible clinical strain | [3] |
| BPY285 |  | Azole-resistant clinical strain | [3] |
| BPY2608 | Related to BPY2813 | Azole-susceptible clinical strain | This study |
| BPY2813 |  | Azole-resistant clinical strain | This study |
| BPY2204 | Related to BPY2212, BPY2240, BPY2254 and BPY2269 | Azole-susceptible clinical strain | This study |
| BPY2212 |  | Azole-susceptible clinical strain | This study |
| BPY2240 |  | Azole-resistant clinical strain | This study |
| BPY2254 |  | Azole-resistant clinical strain | This study |
| BPY2269 |  | Azole-resistant clinical strain | This study |
| Unrelated clinical isolates | | | |
| DSY710 |  | Azole-susceptible clinical strain | This study |
| DSY713 |  | Azole-susceptible clinical strain | This study |
| DSY3632 |  | Azole-susceptible clinical strain | This study |
| DSY3633 |  | Azole-susceptible clinical strain | This study |
| DSY3634 |  | Azole-susceptible clinical strain | This study |
| DSY3635 |  | Azole-susceptible clinical strain | This study |
| DSY3647 |  | Azole-susceptible clinical strain | This study |
| DSY3648 |  | Azole-susceptible clinical strain | This study |
| DSY3649 |  | Azole-susceptible clinical strain | This study |
| DSY3672 |  | Azole-susceptible clinical strain | This study |
| DSY3673 |  | Azole-susceptible clinical strain | This study |
| DSY3680 |  | Azole-susceptible clinical strain | This study |
| DSY3769 |  | Azole-susceptible clinical strain | This study |
| DSY2239 |  | Azole-resistant clinical strain | This study |
| DSY2248 |  | Azole-resistant clinical strain | This study |
| DSY2319 |  | Azole-resistant clinical strain | This study |
| DSY3636 |  | Azole-resistant clinical strain | This study |
| DSY3637 |  | Azole-resistant clinical strain | This study |
| DSY3638 |  | Azole-resistant clinical strain | This study |
| DSY3639 |  | Azole-resistant clinical strain | This study |
| DSY3640 |  | Azole-resistant clinical strain | This study |
| DSY3642 |  | Azole-resistant clinical strain | This study |
| DSY3643 |  | Azole-resistant clinical strain | This study |
| DSY3644 |  | Azole-resistant clinical strain | This study |
| DSY3650 |  | Azole-resistant clinical strain | This study |
| DSY3674 |  | Azole-resistant clinical strain | [1] |
| DSY3675 |  | Azole-resistant clinical strain | This study |
| DSY3676 |  | Azole-resistant clinical strain | This study |
| DSY3677 |  | Azole-resistant clinical strain | This study |
| DSY3678 |  | Azole-resistant clinical strain | This study |
| DSY3679 |  | Azole-resistant clinical strain | This study |
| DSY3770 |  | Azole-resistant clinical strain | This study |
| BPY43 |  | Azole-resistant clinical strain | [3] |
| BPY44 |  | Azole-resistant clinical strain | [3] |
| BPY45 |  | Azole-resistant clinical strain | [3] |
| BPY46 |  | Azole-resistant clinical strain | [3] |
| BPY47 |  | Azole-resistant clinical strain | [3] |
| BPY50 |  | Azole-resistant clinical strain | [3] |
| BPY54 |  | Azole-resistant clinical strain | [3] |
| BPY55 |  | Azole-resistant clinical strain | [1] |
| BPY60 |  | Azole-resistant clinical strain | [3] |
| BPY233 |  | Azole-resistant clinical strain | [3] |
| BPY269 |  | Azole-resistant clinical strain | [1] |
| BPY498 |  | Azole-resistant clinical strain | [1] |
| BPY597 |  | Azole-resistant clinical strain | [1] |
| BPY761 |  | Azole-resistant clinical strain | [1] |
| BPY815 |  | Azole-resistant clinical strain | [1] |
| BPY1068 |  | Azole-resistant clinical strain | [1] |
| BPY1071 |  | Azole-resistant clinical strain | [1] |
| BPY1223 |  | Azole-resistant clinical strain | [1] |
| BPY1565 |  | Azole-resistant clinical strain | [1] |
| BPY2042 |  | Azole-resistant clinical strain | This study |
| BPY2096 |  | Azole-resistant clinical strain | [1] |
| BPY2186 |  | Azole-resistant clinical strain | [1] |
| BPY2394 |  | Azole-resistant clinical strain | [1] |
| Engineered strains | | | |
| SFY53 (DSY562 *pdr1*∆) | DSY562 ura3 | *pdr1∆::SAT1-FLIP* | [1] |
| SFY72 | SFY53 | Contains pSF18 (expression plasmid of *CgPDR1* from DSY2235 | This study |
| SFY73 | SFY53 | Contains pSF19 (expression plasmid of *CgPDR1* from DSY2234) | This study |
| SFY92 | DSY562 | *pdr1*Δ::*SAT1-FLIP* | This study |
| SFY93 | SFY92 | *pdr1*Δ::*FRT* | This study |
| SFY94 | DSY565 | *pdr1*Δ::*SAT1-FLIP* | This study |
| SFY95 | SFY94 | *pdr1*Δ::*FRT* | This study |
| SFY98 | SFY93 | *pdr1*Δ::*CgPDR1-SAT1* (wild-type allele from DSY486) | This study |
| SFY99 | SFY93 | *pdr1*Δ::*CgPDR1-SAT1* (allele from DSY489 with L328F substitution) | This study |
| SFY100 | SFY93 | *pdr1*Δ::*CgPDR1-SAT1* (wild-type allele from DSY738) | This study |
| SFY101 | SFY93 | *pdr1*Δ::*CgPDR1-SAT1* (allele from DSY739 with R376W substitution) | This study |
| SFY102 | SFY93 | *pdr1*Δ::*CgPDR1-SAT1* (wild-type allele from DSY2253) | This study |
| SFY103 | SFY93 | *pdr1*Δ::*CgPDR1-SAT1* (allele from DSY2254 with D1082G substitution) | This study |
| SFY104 | SFY93 | *pdr1*Δ::*CgPDR1-SAT1* (wild-type allele from DSY2235) | This study |
| SFY105 | SFY93 | *pdr1*Δ::*CgPDR1-SAT1* (allele from DSY2234 with T588A substitution) | This study |
| SFY106 | SFY93 | *pdr1*Δ::*CgPDR1-SAT1* (wild-type allele from DSY701) | This study |
| SFY107 | SFY93 | *pdr1*Δ::*CgPDR1-SAT1* (allele from DSY704 with T607S substitution) | This study |
| SFY108 | SFY93 | *pdr1*Δ::*CgPDR1-SAT1* (wild-type allele from DSY529) | This study |
| SFY109 | SFY93 | *pdr1*Δ::*CgPDR1-SAT1* (allele from DSY530 with E1083Q substitution) | This study |
| SFY110 | SFY93 | *pdr1*Δ::*CgPDR1-SAT1* (wild-type allele from DSY753) | This study |
| SFY111 | SFY93 | *pdr1*Δ::*CgPDR1-SAT1* (allele from DSY754 with Y584C substitution) | This study |
| SFY112 | SFY93 | *pdr1*Δ::*CgPDR1-SAT1* (wild-type allele from DSY726) | This study |
| SFY113 | SFY93 | *pdr1*Δ::*CgPDR1-SAT1* (allele from DSY727 with D876Y substitution) | This study |
| SFY114 | SFY93 | *pdr1*Δ::*CgPDR1-SAT1* (wild-type allele from DSY562) | This study |
| SFY115 | SFY93 | *pdr1*Δ::*CgPDR1-SAT1* (allele from DSY565 with L280F substitution) | This study |
| SFY116 | SFY93 | *pdr1*Δ::*CgPDR1-SAT1* (allele from BPY55 with P822L substitution) | This study |
| SFY118 | SFY95 | *pdr1*Δ::*CgPDR1-SAT1* (wild-type allele from DSY562) | This study |
| SFY119 | SFY95 | *pdr1*Δ::*CgPDR1-SAT1* (allele from DSY565 with L280F substitution) | This study |
| SFY120 | SFY95 | *pdr1*Δ::*CgPDR1-SAT1* (allele from BPY55 with P822L substitution) | This study |

**References**

1. Torelli R, Posteraro B, Ferrari S, La Sorda M, Fadda G, et al. (2008) The ATP-binding cassette transporter-encoding gene *CgSNQ2* is contributing to the *CgPDR1*-dependent azole resistance of *Candida glabrata*. Mol Microbiol 68: 186-201.

2. Sanglard D, Ischer F, Calabrese D, Majcherczyk PA, Bille J (1999) The ATP binding cassette transporter gene *CgCDR1* from *Candida glabrata* is involved in the resistance of clinical isolates to azole antifungal agents. Antimicrob Agents Chemother 43: 2753-2765.

3. Sanguinetti M, Posteraro B, Fiori B, Ranno S, Torelli R, et al. (2005) Mechanisms of azole resistance in clinical isolates of *Candida glabrata* collected during a hospital survey of antifungal resistance. Antimicrob Agents Chemother 49: 668-679.
