# Supplementary material for: Gain of Function Mutations in CgPDR1 of Candida glabrata Not Only Mediate Antifungal Resistance but Also Enhance Virulence
Source: PLoS Pathog. 2009 Jan 16;5(1):e1000268. doi: 10.1371/journal.ppat.1000268 (PMC2607542; doi:10.1371/journal.ppat.1000268)
Supplement: Table S3 — Details of statistical analysis. (0.27 MB DOC) [file ppat.1000268.s008.doc]

**Table S3: Details of statistical analysis**

# A. Fluconazole therapy

| **Comparison** | **Tissue burden (geometric mean)** | ***P* value ^a)^** | **Significance** |
| --- | --- | --- | --- |
|  |  |  |  |
| ***Kidney*** |  |  |  |
| DSY562 vs DSY562 treated | 1.126e+007 vs 3.956e+005 | <0.0001 | S*** |
| DSY565 vs DSY565 treated | 9.532e+007 vs 5.664e+007 | 0.04 | S* |
| DSY565 pdr1Δ vs DSY565 pdr1Δ treated | 2.604e+006 vs 2.902e+003 | <0.0001 | S*** |
| DSY562 pdr1Δ vs DSY562 pdr1Δ treated | 4.936e+006 vs 4.283e+003 | <0.0001 | S*** |
| DSY562 pdr1Δ L280F vs DSY562 pdr1Δ L280F treated | 4.436e+007 vs 3.602e+007 | 0.76 | NS |
| DSY565 pdr1Δ PDR1 vs DSY565 pdr1Δ PDR1 treated | 5.109e+006 vs 3.384e+005 | <0.0001 | S*** |
| DSY562 pdr1Δ PDR1 vs DSY562 pdr1Δ PDR1 treated | 5.256e+006 vs 7.309e+005 | 0.0002 | S*** |
| DSY565 pdr1Δ L280F vs DSY565 pdr1Δ L280F treated | 1.302e+008 vs 3.188e+007 | 0.003 | S** |
| DSY562 pdr1Δ T588A vs DSY562 pdr1Δ T588A treated | 8.120e+007 vs 1.437e+007 | 0.015 | S* |
| DSY562 pdr1Δ E1083Q vs DSY562 pdr1Δ E1083Q treated | 7.514e+007 vs 4.351e+007 | 0.14 | NS |
| DSY562 pdr1Δ P822L vs DSY562 pdr1Δ P822L treated | 2.076e+008 vs 1.075e+008 | 0.07 | NS |
| DSY565 pdr1Δ P822L vs DSY565 pdr1Δ P822L treated | 1.147e+008 vs 6.442e+007 | 0.14 | NS |
| DSY562 vs DSY562 pdr1Δ treated | 1.126e+007 vs 2.604e+006 | <0.0001 | S*** |
| DSY565 vs DSY565 pdr1Δ treated | 9.532e+007 vs 4.936e+006 | <0.0001 | S*** |
| DSY562 vs DSY562 pdr1Δ L280F treated | 1.126e+007 vs 3.602e+007 | <0.0001 | S** |
| DSY565 vs DSY565 pdr1Δ PDR1 treated | 9.532e+007 vs 3.384e+005 | 0.0015 | S** |
| DSY562 treated vs DSY562 pdr1Δ treated | 1.126e+007 vs 4.283e+003 | <0.0001 | S*** |
| DSY565 treated vs DSY565 pdr1Δ treated | 9.532e+007 vs 2.902e+003 | 0.0002 | S*** |
| DSY562 vs DSY562 pdr1Δ PDR1 treated | 1.126e+007 vs 7.309e+005 | <0.0001 | S*** |
| DSY565 vs DSY565 pdr1Δ L280F treated | 9.532e+007 vs 3.188e+007 | 0.0015 | S** |
|  |  |  |  |
| ***Spleen*** |  |  |  |
| DSY562 vs DSY562 treated | 3.380e+005 vs 1.261e+005 | 0.028 | S* |
| DSY565 vs DSY565 treated | 5.926e+006 vs 3.657e+006 | 0.39 | NS |
| DSY565 pdr1Δ vs DSY565 pdr1Δ treated | 1.894e+005 vs 3.152e+003 | 0.0002 | S*** |
| DSY562 pdr1Δ vs DSY562 pdr1Δ treated | 2.284e+005 vs 3.951e+003 | <0.0001 | S*** |
| DSY562 pdr1Δ L280F vs DSY562 pdr1Δ L280F treated | 1.945e+006 vs 9.342e+005 | 0.43 | NS |
| DSY565 pdr1Δ PDR1 vs DSY565 pdr1Δ PDR1 treated | 5.926e+006 vs 3.657e+006 | 0.12 | NS |
| DSY562 pdr1Δ PDR1 vs DSY562 pdr1Δ PDR1 treated | 2.047e+005 vs 4.563e+004 | 0.0005 | S*** |
| DSY565 pdr1Δ L280F vs DSY565 pdr1Δ L280F treated | 1.292e+007 vs 4.249e+006 | 0.015 | S* |
| DSY562 pdr1Δ T588A vs DSY562 pdr1Δ T588A treated | 5.917e+006 vs 3.158e+006 | 0.28 | NS |
| DSY562 pdr1Δ E1083Q vs DSY562 pdr1Δ E1083Q treated | 8.408e+006 vs 6.823e+006 | 0.91 | NS |
| DSY562 pdr1Δ P822L vs DSY562 pdr1Δ P822L treated | 3.245e+006 vs 4.770e+006 | 0.63 | NS |
| DSY565 pdr1Δ P822L vs DSY565 pdr1Δ P822L treated | 1.043e+006 vs 7.798e+005 | 0.34 | NS |
| DSY562 vs DSY562 pdr1Δ treated | 3.380e+005 vs 3.951e+003 | <0.0001 | S*** |
| DSY565 vs DSY565 pdr1Δ treated | 5.926e+006 vs 3.152e+003 | 0.0002 | S*** |
| DSY562 vs DSY562 pdr1Δ L280F treated | 3.380e+005 vs 9.342e+005 | <0.0001 | S*** |
| DSY565 vs DSY565 pdr1Δ PDR1 treated | 5.926e+006 vs 3.657e+006 | 0.0002 | S*** |
| DSY562 treated vs DSY562 pdr1Δ treated | 1.261e+005 vs 4.283e+003 | 0.0002 | S*** |
| DSY565 treated vs DSY565 pdr1Δ treated | 5.664e+006 vs 2.902e+003 | <0.0001 | S*** |
| DSY562 vs DSY562 pdr1Δ PDR1 treated | 3.380e+005 vs 4.563e+004 | <0.0001 | S*** |
| DSY565 vs DSY565 pdr1Δ L280F treated | 5.926e+006 vs 4.249e+006 | 0.47 | NS |
|  |  |  |  |
| ***Liver*** |  |  |  |
| DSY562 vs DSY562 treated | 3.207e+004 vs 3.295e+003 | <0.0001 | S*** |
| DSY565 vs DSY565 treated | 2.088e+005 vs 2.551e+005 | 0.82 | NS |
| DSY565 pdr1Δ vs DSY565 pdr1Δ treated | 4.680e+004 vs 3.240e+002 | <0.0001 | S*** |
| DSY562 pdr1Δ vs DSY562 pdr1Δ treated | 1.199e+004 vs 2.640e+002 | <0.0001 | S*** |
| DSY562 pdr1Δ L280F vs DSY562 pdr1Δ L280F treated | 9.512e+004 vs 1.824e+005 | 0.05 | S* |
| DSY565 pdr1Δ PDR1 vs DSY565 pdr1Δ PDR1 treated | 1.453e+004 vs 2.755e+003 | 0.0003 | S*** |
| DSY562 pdr1Δ PDR1 vs DSY562 pdr1Δ PDR1 treated | 2.535e+004vs 5.868e+003 | 0.009 | S** |
| DSY565 pdr1Δ L280F vs DSY565 pdr1Δ L280F treated | 2.381e+005 vs 25182e+005 | 1.0 | NS |
| DSY562 pdr1Δ T588A vs DSY562 pdr1Δ T588A treated | 2.808e+005 vs 1.291e+005 | 0.16 | NS |
| DSY562 pdr1Δ E1083Q vs DSY562 pdr1Δ E1083Q treated | 1.834e+005 vs 8.515e+004 | 0.27 | NS |
| DSY562 pdr1Δ P822L vs DSY562 pdr1Δ P822L treated | 4.110e+005 vs1.631e+005 | 0.03 | S* |
| DSY565 pdr1Δ P822L vs DSY565 pdr1Δ P822L treated | 7.497e+005 vs3.473e+005 | 0.24 | NS |
| DSY562 vs DSY562 pdr1Δ treated | 3.207e+004 vs 2.640e+002 | <0.0001 | S*** |
| DSY562 vs DSY562 pdr1Δ treated | 3.207e+004 vs 2.640e+002 | <0.0001 | S*** |
| DSY565 vs DSY565 pdr1Δ treated | 2.088e+005 vs 3.240e+002 | <0.0001 | S*** |
| DSY562 vs DSY562 pdr1Δ L280F treated | 3.207e+004 vs 1.824e+005 | 0.005 | S** |
| DSY565 vs DSY565 pdr1Δ PDR1 treated | 2.088e+005 vs 3.240e+002 | <0.0001 | S*** |
| DSY562 treated vs DSY562 pdr1Δ treated | 3.295e+003vs 2.640e+002 | 0.0002 | S*** |
| DSY565 treated vs DSY565 pdr1Δ treated | 2.551e+005 vs 3.240e+002 | <0.0001 | S*** |
| DSY562 vs DSY562 pdr1Δ PDR1 treated | 3.207e+004 vs 5.868e+003 | 0.003 | S** |
| DSY565 vs DSY565 pdr1Δ L280F treated | 2.088e+005 vs 2.518e+005 | 0.73 | NS |

* *P*<0.05; ** *P*<0.01; *** *P*<0.001

**B. Virulence immuno-suppressed**

| **Comparison** | **Tissue burden (geometric mean)** | ***P* value** | **Significance** |
| --- | --- | --- | --- |
|  |  |  |  |
| ***Kidney*** |  |  |  |
| DSY562 vs DSY565 | 6.648e+006 vs 4.050e+007 | 0.0007 | S*** |
| DSY562 vs DSY562 pdr1Δ | 6.648e+006 vs 5.237e+006 | 0.79 | NS |
| DSY562 vs DSY562 pdr1Δ PDR1 | 6.648e+006 vs 1.997e+006 | 0.03 | S* |
| DSY562 vs DSY562 pdr1Δ L280F | 6.648e+006 vs 2.100e+007 | 0.003 | S** |
| DSY562 vs DSY562 pdr1Δ T588A | 6.648e+006 vs 4.116e+007 | 0.003 | S** |
| DSY562 vs DSY562 pdr1Δ E1083Q | 6.648e+006 vs 3.812e+007 | 0.002 | S** |
| DSY562 vs DSY565 pdr1Δ L280F | 6.648e+006 vs 1.302e+008 | <0.0001 | S*** |
| DSY562 vs DSY562 pdr1Δ P822L | 6.648e+006 vs 5.596e+007 | <0.0001 | S*** |
| DSY565 vs DSY565 pdr1Δ | 4.050e+007 vs 2.604e+006 | <0.0001 | S*** |
| DSY565 vs DSY565 pdr1Δ L280F | 4.050e+007 vs 6.687e+007 | 0.73 | NS |
| DSY565 vs DSY565 pdr1Δ PDR1 | 4.050e+007 vs 2.595e+006 | <0.0001 | S*** |
| DSY565 vs DSY565 pdr1Δ P822L | 4.050e+007 vs 1.034e+008 | 0.12 | NS |
| DSY562 pdr1Δ L280F vs DSY565 pdr1Δ PDR1 | 4.436e+007 vs 2.595e+006 | <0.0001 | S*** |
| DSY562 vs DSY565 pdr1Δ PDR1 | 6.648e+006 vs 2.595e+006 | 0.03 | S* |
| DSY562 pdr1Δ PDR1 vs DSY565 pdr1Δ PDR1 | 1.997e+006 vs 2.595e+006 | 0.24 | NS |
| DSY562 pdr1Δ PDR1 vs DSY565 pdr1Δ L280F | 1.997e+006 vs 6.687e+007 | 0.0002 | S*** |
|  |  |  |  |
| ***Spleen*** |  |  |  |
| DSY562 vs DSY565 | 3.380e+005 vs 5.926e+006 | <0.0001 | S*** |
| DSY562 vs DSY562 pdr1Δ | 3.380e+005 vs 2.284e+005 | 0.16 | NS |
| DSY562 vs DSY562 pdr1Δ PDR1 | 3.380e+005 vs 2.047e+005 | 0.19 | NS |
| DSY562 vs DSY562 pdr1Δ L280F | 3.380e+005 vs 1.945e+006 | <0.0001 | S*** |
| DSY562 vs DSY562 pdr1Δ T588A | 3.380e+005 vs 5.917e+006 | <0.0001 | S*** |
| DSY562 vs DSY562 pdr1Δ E1083Q | 3.380e+005 vs 8.408e+006 | <0.0001 | S*** |
| DSY562 vs DSY565 pdr1Δ L280F | 3.380e+005 vs 1.292e+007 | <0.0001 | S*** |
| DSY562 vs DSY562 pdr1Δ P822L | 3.380e+005 vs 1.043e+006 | <0.0001 | S*** |
| DSY565 vs DSY565 pdr1Δ | 5.926e+006 vs 1.894e+005 | 0.12 | NS |
| DSY565 vs DSY565 pdr1Δ L280F | 5.926e+006 vs 1.292e+007 | 0.97 | NS |
| DSY565 vs DSY565 pdr1Δ PDR1 | 5.926e+006 vs 2.172e+005 | 0.0002 | S*** |
| DSY565 vs DSY565 pdr1Δ P822L | 5.926e+006 vs 3.245e+006 | 0.24 | NS |
| DSY562 pdr1Δ L280F vs DSY565 pdr1Δ PDR1 | 1.945e+006 vs 2.172e+005 | 0.002 | S** |
| DSY562 vs DSY565 pdr1Δ PDR1 | 2.047e+005 vs 2.172e+005 | 0.24 | NS |
| DSY562 pdr1Δ PDR1 vs DSY565 pdr1Δ PDR1 | 2.047e+005 vs 2.172e+005 | 0.68 | NS |
| DSY562 pdr1Δ PDR1 vs DSY565 pdr1Δ L280F | 2.047e+005 vs 1.292e+007 | <0.0001 | S*** |
|  |  |  |  |
| ***Liver*** |  |  |  |
| DSY562 vs DSY565 | 3.207e+004 vs 2.415e+004 | <0.0001 | S*** |
| DSY562 vs DSY562 pdr1Δ | 3.207e+004 vs 1.199e+004 | 0.009 | * |
| DSY562 vs DSY562 pdr1Δ PDR1 | 3.207e+004 vs 2.535e+004 | 0.63 | NS |
| DSY562 vs DSY562 pdr1Δ L280F | 3.207e+004 vs 9.511e+004 | 0.0052 | S** |
| DSY562 vs DSY562 pdr1Δ T588A | 3.207e+004 vs 2.808e+005 | 0.0001 | S*** |
| DSY562 vs DSY562 pdr1Δ E1083Q | 3.207e+004 vs 1.834e+005 | 0.0005 | S*** |
| DSY562 vs DSY565 pdr1Δ L280F | 3.207e+004 vs 2.381e+005 | <0.0001 | S*** |
| DSY562 vs DSY562 pdr1Δ P822L | 3.207e+004 vs 4.110e+005 | <0.0001 | S*** |
| DSY565 vs DSY565 pdr1Δ | 2.415e+004 vs 4.680e+004 | <0.0001 | S*** |
| DSY565 vs DSY565 pdr1Δ L280F | 2.415e+004 vs 2.381e+005 | 0.85 | NS |
| DSY565 vs DSY565 pdr1Δ PDR1 | 2.415e+004 vs 1.453e+004 | <0.0001 | S*** |
| DSY565 vs DSY565 pdr1Δ P822L | 2.415e+004 vs 7.497e+005 | 0.0145 | S* |
| DSY562 pdr1Δ L280F vs DSY565 pdr1Δ PDR1 | 9.511e+004 vs 1.453e+004 | 0.0003 | S*** |
| DSY562 vs DSY565 pdr1Δ PDR1 | 3.207e+004 vs 1.453e+004 | 0.053 | NS |
| DSY562 pdr1Δ PDR1 vs DSY565 pdr1Δ PDR1 | 2.535e+004 vs 1.453e+004 | 0.12 | NS |
| DSY562 pdr1Δ PDR1 vs DSY565 pdr1Δ L280F | 2.535e+004 vs 2.381e+005 | <0.0001 | S*** |
|  |  |  |  |

* *P*<0.05; ** *P*<0.01; *** *P*<0.001; NS: *P*>0.05

**C. Virulence Immuno-competent**

| **Comparison** | **Tissue burden (geometric mean)** | ***P* value** | **Significance** |
| --- | --- | --- | --- |
|  |  |  |  |
| ***Kidney*** |  |  |  |
| DSY562 vs DSY565 | 1.126e+007 vs 9.532e+007 | <0.0001 | S*** |
| DSY562 vs DSY562 pdr1Δ | 1.126e+007 vs 4.936e+006 | 0.07 | NS |
| DSY562 vs DSY562 pdr1Δ PDR1 | 1.126e+007 vs 5.109e+006 | 0.07 | NS |
| DSY562 vs DSY562 pdr1Δ L280F | 1.126e+007 vs 4.436e+007 | <0.0001 | S*** |
| DSY562 vs DSY562 pdr1Δ T588A | 1.126e+007 vs 8.120e+007 | 0.002 | S** |
| DSY562 vs DSY562 pdr1Δ E1083Q | 1.126e+007 vs 7.514e+007 | 0.001 | S** |
| DSY562 vs DSY565 pdr1Δ L280F | 1.126e+007 vs 1.302e+008 | <0.0001 | S*** |
| DSY562 vs DSY562 pdr1Δ P822L | 1.126e+007 vs 1.147e+008 | <0.0001 | S*** |
| DSY565 vs DSY565 pdr1Δ | 9.532e+007 vs 2.604e+006 | <0.0001 | S*** |
| DSY565 vs DSY565 pdr1Δ L280F | 9.532e+007 vs 4.436e+007 | 0.97 | NS |
| DSY565 vs DSY565 pdr1Δ PDR1 | 9.532e+007 vs 5.109e+006 | <0.0001 | S*** |
| DSY565 vs DSY565 pdr1Δ P822L | 9.532e+007 vs 2.076e+008 | 0.16 | NS |
| DSY562 pdr1Δ L280F vs DSY565 pdr1Δ PDR1 | 4.436e+007 vs 5.109e+006 | <0.0001 | S*** |
| DSY562 vs DSY565 pdr1Δ PDR1 | 1.126e+007 vs 5.109e+006 | 0.05 | S* |
| DSY562 pdr1Δ PDR1 vs DSY565 pdr1Δ PDR1 | 5.256e+006 vs 5.109e+006 | 0.62 | NS |
| DSY562 pdr1Δ PDR1 vs DSY565 pdr1Δ L280F | 5.256e+006 vs 1.302e+008 | 0.0002 | S*** |
|  |  |  |  |
| ***Spleen*** |  |  |  |
| DSY562 vs DSY565 | 1.755e+005 vs 3.172e+006 | <0.0001 | S** |
| DSY562 vs DSY562 pdr1Δ | 1.755e+005 vs 1.229e+005 | 0.35 | NS |
| DSY562 vs DSY562 pdr1Δ PDR1 | 1.755e+005 vs 1.103e+005 | 0.31 | NS |
| DSY562 vs DSY562 pdr1Δ L280F | 1.755e+005 vs 1.028e+006 | <0.0001 | S*** |
| DSY562 vs DSY562 pdr1Δ T588A | 1.755e+005 vs 2.509e+006 | <0.0001 | S*** |
| DSY562 vs DSY562 pdr1Δ E1083Q | 1.755e+005 vs 4.525e+006 | <0.0001 | S*** |
| DSY562 vs DSY565 pdr1Δ L280F | 1.755e+005 vs 6.927e+006 | <0.0001 | S*** |
| DSY562 vs DSY562 pdr1Δ P822L | 1.755e+005 vs 1.033e+006 | <0.0001 | S*** |
| DSY565 vs DSY565 pdr1Δ | 3.172e+006 vs 1.066e+005 | 0.0002 | S*** |
| DSY565 vs DSY565 pdr1Δ L280F | 3.172e+006 vs 6.927e+006 | 0.30 | NS |
| DSY565 vs DSY565 pdr1Δ PDR1 | 3.172e+006 vs 0.926e+005 | 0.0002 | S*** |
| DSY565 vs DSY565 pdr1Δ P822L | 3.172e+006 vs 1.746e+006 | 0.34 | NS |
| DSY562 pdr1Δ L280F vs DSY565 pdr1Δ PDR1 | 1.028e+006 vs 0.926e+005 | <0.0001 | S*** |
| DSY562 vs DSY565 pdr1Δ PDR1 | 1.755e+005 vs 0.926e+005 | 0.10 | NS |
| DSY562 pdr1Δ PDR1 vs DSY565 pdr1Δ PDR1 | 1.103e+005 vs 0.926e +005 | 0.48 | NS |
| DSY562 pdr1Δ PDR1 vs DSY565 pdr1Δ L280F | 1.103e+005 vs 6.927e+006 | <0.0001 | S*** |
|  |  |  |  |
| ***Liver*** |  |  |  |
| DSY562 vs DSY565 | 1.552e+004 vs 1.229e+005 | <0.0001 | S*** |
| DSY562 vs DSY562 pdr1Δ | 1.552e+004 vs 9.834e+003 | 0.21 | NS |
| DSY562 vs DSY562 pdr1Δ PDR1 | 1.552e+004 vs 1.226e+004 | 0.52 | NS |
| DSY562 vs DSY562 pdr1Δ L280F | 1.552e+004 vs 4.823e+004 | 0.003 | S** |
| DSY562 vs DSY562 pdr1Δ T588A | 1.552e+004 vs 1.434e+005 | 0.0001 | S*** |
| DSY562 vs DSY562 pdr1Δ E1083Q | 1.552e+004 vs 9.150e+004 | 0.0003 | S*** |
| DSY562 vs DSY565 pdr1Δ L280F | 1.552e+004 vs 1.184e+005 | <0.0001 | S*** |
| DSY562 vs DSY562 pdr1Δ P822L | 1.552e+004 vs 1.939e+005 | <0.0001 | S*** |
| DSY565 vs DSY565 pdr1Δ | 1.229e+005 vs 1.826e+004 | <0.0001 | S*** |
| DSY565 vs DSY565 pdr1Δ L280F | 1.229e+005 vs 1.184e+005 | 1.00 | NS |
| DSY565 vs DSY565 pdr1Δ PDR1 | 1.229e+005 vs 7.169e+003 | <0.0001 | S*** |
| DSY565 vs DSY565 pdr1Δ P822L | 1.229e+005 vs 2.978e+005 | 0.06 | NS |
| DSY562 pdr1Δ L280F vs DSY565 pdr1Δ PDR1 | 4.823e+004 vs 7.169e+003 | 0.0005 | S*** |
| DSY562 vs DSY565 pdr1Δ PDR1 | 1.552e+004 vs 7.169e+003 | 0.04 | S* |
| DSY562 pdr1Δ PDR1 vs DSY565 pdr1Δ PDR1 | 1.226e+004 vs 7.169e+003 | 0.09 | NS |
| DSY562 pdr1Δ PDR1 vs DSY565 pdr1Δ L280F | 1.226e+004 vs 1.184e+005 | <0.0001 | S*** |

Symbols :

^a)^ : The reported statistical values are originating from data shown in Fig. 7, 10, S4 and S5 and used non-parametric Wilocoxon Rank sum tests.

* *P*<0.05; ** *P*<0.01; *** *P*<0.001; NS: *P*>0.05
